# Diminazene aceturate mitigates cardiomyopathy by interfering with renin-angiotensin system in a septic rat model

**DOI:** 10.1186/s40360-022-00584-4

**Published:** 2022-07-04

**Authors:** Zhaoqing Lu, Di Wu, Zheng Wang, Hanyu Zhang, Yufan Du, Guoxing Wang

**Affiliations:** Department of Emergency Medicine, Beijing Friendship Hospital, Capital Medical University, Beijing, 100050 China

**Keywords:** Septic cardiomyopathy, Diminazene aceturate, Angiotensin-converting enzyme, Renin-angiotensin system

## Abstract

**Background:**

There were limited studies investigating treatments of septic cardiomyopathy (SCM), which is a common complication during sepsis. A septic rat model created by cecal ligation and puncture (CLP) was used to investigate the effects of diminazene aceturate (DIZE) in SCM.

**Methods:**

A total of 151 Wistar rats were randomly assigned into the sham, CLP, or CLP + DIZE group. Data evaluated postoperatively at 6, 12, 24, and 48 hours included: cardiac function; plasma concentrations of tumor necrosis factor-alpha (TNF-α), interleukin-6, angiotensin-(1–7) [Ang-(1–7)], angiotensin II (AngII), troponin I, and brain natriuretic peptide; expression levels of myocardial Ang-(1–7), angiotensin-converting enzyme (ACE), ACE2, and angiotensin type 1 and Mas receptors; and histological changes.

**Results:**

We found that the CLP + DIZE group had a lower mortality compared to the CLP group (38.5% versus 61.5%) within 48 h postoperatively, although without statistical significance. In contrast to the sham group, the CLP group had decreased cardiac functions, increased myocardial injuries, and higher TNF-α levels, which were ameliorated in the CLP + DIZE group. Furthermore, administration of DIZE could reverse the decreases of myocardial Ang-(1–7) and ACE2 expressions in the CLP group, which finally minimized the myocardial microstructure disruptions.

**Conclusions:**

It was concluded that DIZE could mitigate the development of SCM and preserve cardiac function during sepsis possibly by interfering with the renin-angiotensin system through promoting myocardial ACE2 expression and restoring local Ang-(1–7) levels.

**Supplementary Information:**

The online version contains supplementary material available at 10.1186/s40360-022-00584-4.

## Background

Sepsis is a dysregulated systemic response to infection and can be lethal without appropriate treatments [1]. During sepsis, various chemicals and cytokines are released to fight the infection, but these chemicals and cytokines also cause a cascade of reactions that lead to multiple organ dysfunction [2]. Various systems can be affected during the process of sepsis. Heart is reported to be one of the most frequently affected organs [3]. In the 1980s, septic cardiomyopathy (SCM) was first described as an acutely depressed left ventricular ejection fraction with ventricular dilation that occurred during sepsis [4]. In patients with sepsis, the prevalence of SCM has been reported to vary between 10 and 70% [4], and septic patients with SCM have an increased mortality rate of up to 70–90% [5]. Although there are several guidelines with recommendations to treat patients with sepsis [6, 7], there is currently no effective treatment available to prevent the development of or directly treat SCM in septic patients.

The renin-angiotensin system (RAS) participates in the pathophysiology of cardiovascular disorders [8]. RAS includes two counteracting pathways: (1) the angiotensin-converting enzyme (ACE) /angiotensin (Ang) II/angiotensin type 1 receptor (AT1R) axis, and (2) the ACE2/Ang-(1–7)/Mas receptor (MasR) axis. Interventions involving ACE2 and Ang-(1–7) are the key factors involved in protection from cardiovascular diseases, particularly heart failure [9]. Agents that target the RAS pathway might serve as a novel therapeutic strategy to prevent patients from developing SCM during sepsis.

Diminazene aceturate (DIZE) is the most common therapeutic medication to treat trypanosomiasis in domestic animals [10], and it has also been used to treat trypanosomiasis in humans [11]. The main mechanism of action involves inhibitions of DNA duplication and mitochondrial type II topoisomerases [12]. In addition, it can also enhance the conversion of ACE2-mediated angiotensin [13]. Previous experiments showed that DIZE could prevent the development of pulmonary hypertension by stimulating the vasoprotective axis of the RAS and improve cardiac function by lowering levels of inflammatory cytokines [14–16]. While the ACE2/Ang-(1–7)/MasR axis has been studied in animal models of sepsis as a therapeutic target [17–19], the ability of DIZE to affect this axis and attenuate the development of SCM is unknown.

In the present study, DIZE was used as an ACE2 activator to investigate whether DIZE could prevent the development of SCM in a rat model of sepsis. The potential involvement of the ACE2/Ang-(1–7)/MasR axis was also studied.

## Methods

### Experimental animals and ethical approval

The present study was conducted using 151 male Wistar rats (10–11 weeks old, 280–320 g) obtained from Beijing Charles River Animal Experiment Technology Co. Ltd. The pathogen-free rats were hosted in the animal center at Beijing Friendship Hospital in Beijing, China, with access to food and water ad libitum, under a 12-hour light/dark cycle, and at a temperature of 20–23 °C. This study was approved by the Animal Research Ethics Committee of Beijing Friendship Hospital (Certification no. 15–1004). All procedures performed with these animals complied with the ARRIVE guidelines.

### Study protocol

The rats were randomly assigned into either the sham (37 rats), cecal ligation and puncture (CLP; 57 rats), or CLP + DIZE (57 rats) group.

DIZE (Cayman Chemical, Michigan, USA) was dissolved to a final concentration of 2 mg/mL in normal saline. The CLP + DIZE group was injected subcutaneously with the DIZE solution (15 mg/kg) 30 minutes prior to the operation [20], whereas the sham and CLP groups received 7.5 mL/kg normal saline injected subcutaneously.

The rat model of sepsis was created using the CLP method as described previously [21, 22]. Briefly, rats were fasted for 24 hours. Then, sodium pentobarbital (50 mg/kg; Sinopharm Chemical Reagent Co. Ltd.) was injected intraperitoneally for anesthesia. After securing and shaving the rats, an incision (approximately 2 cm) was made on the anterior midline abdomen to expose the cecum. The cecum was ligated 1.0 cm from the tip. To induce sepsis, before the abdominal wall was closed, an 18-gauge injection needle was used to puncture the ligated end of the cecum two times, which allowed fecal material to extrude into the abdominal cavity. A postoperative subcutaneous injection of warm normal saline (20 mL/kg) was given for resuscitation. The rats in the sham group received open abdominal surgery but with no ligation or perforation of the cecum.

### Survival analysis

In each group, 13 rats were observed for 48 hours after the surgical procedure to evaluate survival.

### Cardiac function assessments

Cardiac function was assessed postoperatively as previously described [23]. Briefly, in each group, six rats per timepoint were sacrificed at 6, 12, 24 and 48 hours post-surgery to evaluate cardiac function. Following pentobarbital (50 mg/kg) anesthesia, a polyurethane catheter was introduced through the right common carotid artery into the left ventricle. Hemodynamic measurements, including the left ventricular systolic pressure (LVSP), maximal left ventricular pressure rising rate (+dp/dtmax), and maximal left ventricular pressure declining rate (−dp/dtmax) were measured and documented using an eight-channel polygraph system (AD Instruments PL3508B35, Shanghai, China). Data were analyzed using LabChart 8.0 software. Next, blood samples were collected and placed in a tube with heparin. The plasma was isolated and frozen for future analysis. After rats were euthanized with an overdose of pentobarbital, tissue samples from the left ventricle were obtained immediately and were frozen at − 80 °C for western blot analysis or fixed for histological assessments.

### Enzyme-linked immunosorbent assay (ELISA)

Plasma levels of TNF-α, interleukin-6 (IL-6), Ang-(1–7), AngII, troponin I (TnI), and brain natriuretic peptide (BNP) were measured using commercial ELISA kits (Cusabio, Wuhan, China; cat no. CSB-E11987r, CSB-E04640r, CSB-E14241r, CSB-E04494r, CSB-E08594r, and CSB-E07972r, respectively) according to the instructions from the manufacturer. The same rat-specific ELISA kits also used to determine the level of Ang-(1–7) in the cardiac tissue homogenates. The results were presented as picograms per milligram tissue.

### Western blot analysis

Protein expression levels of ACE, ACE2, AT1R, and MasR in cardiac tissue were determined using western blotting as previously described [24]. In brief, frozen cardiac tissues were thawed, ground, and homogenized in the lysis buffer that contained protease inhibitors, surfactants, as well as phosphatase inhibitors. Protein samples were loaded in 8% or 10% gels for sodium dodecyl sulphate-polyacrylamide gel electrophoresis. The resolved proteins were electrically transferred to the nitrocellulose membranes and detected using specific antibodies, including anti-ACE (SantaCruz Biotechnology, Inc., CA, USA; cat no. sc-23,908; lot no. C1319), anti-ACE2 (Abcam, UK; cat no. ab108252; lot no. GR145000–28), anti-AT1R (SantaCruz Biotechnology, Inc., CA, USA; cat no. sc515884; lot no. J0319), and anti-MasR (SantaCruz Biotechnology, Inc., CA, USA; cat no. sc-390,453; lot no. A1419), to quantify myocardial protein levels. The primary antibody-antigen complexes and the horseradish peroxidase (HRP)-conjugated secondary antibodies were incubated and then visualized using an enhanced chemiluminescence (ECL) substrate (Thermo Fisher Scientific Inc., USA) and ECL hyperfilm (GE Healthcare Pvt. Ltd., UK). Blots were inspected by ImageJ software (NIH, USA). Results were normalized with the beta-actin (Sigma-Aldrich, USA; cat no. A5441) as the internal standard.

### Histological evaluations

After sacrificing the rats, the myocardium of the left ventricle was rapidly separated and cleaned with ice-cold normal saline. Then, the tissues were fixed in a 4% paraformaldehyde solution, blocked in paraffin, cut (5 μm thick), and stained with hematoxylin and eosin. The stained sections were inspected under a light microscope. Photos were captured using a Leica fluorescence microscope (DM 2500) at 400× magnification.

### Transmission electron microscopy

Left ventricular myocardial tissues were sunk in 2.5% glutaraldehyde (3 hours) and then in 2% osmium tetroxide (1 hour) for fixation. After staining with uranyl acetate overnight, the tissues were examined under a transmission electron microscope (H-7650, Hitachi High-Technologies Corp., Tokyo, Japan) at 50,000× and 100,000× magnification.

### Statistical analysis

All data were analyzed using SPSS 22.0 software (SPSS, Inc., Chicago, IL, USA). Shapiro-Wilk tests were used to test for normal distribution. Continuous data with normal distribution were presented as the mean ± standard deviation, while continuous data without normal distribution including plasma TNF-α, TnI, BNP, AngII and Ang-(1–7) were log-transformed to meet the normal distribution, and then were analyzed by one-way analysis of variance (ANOVA) combined with the LSD post-hoc test. Data with heteroscedasticity (Bartlett’s test) were analyzed using a nonparametric test (Kruskal-Wallis test followed by Dunn’s multiple comparisons test). Data that are normally *distributed* have been presented in bar graphs, while data without normal distribution have been presented in box plots. Survival distributions were compared by a log-rank test and shown as Kaplan-Meier curves. Statistical significance was considered with a *P* value < 0.05.

## Results

### Survival outcomes

**Fig. 1:**
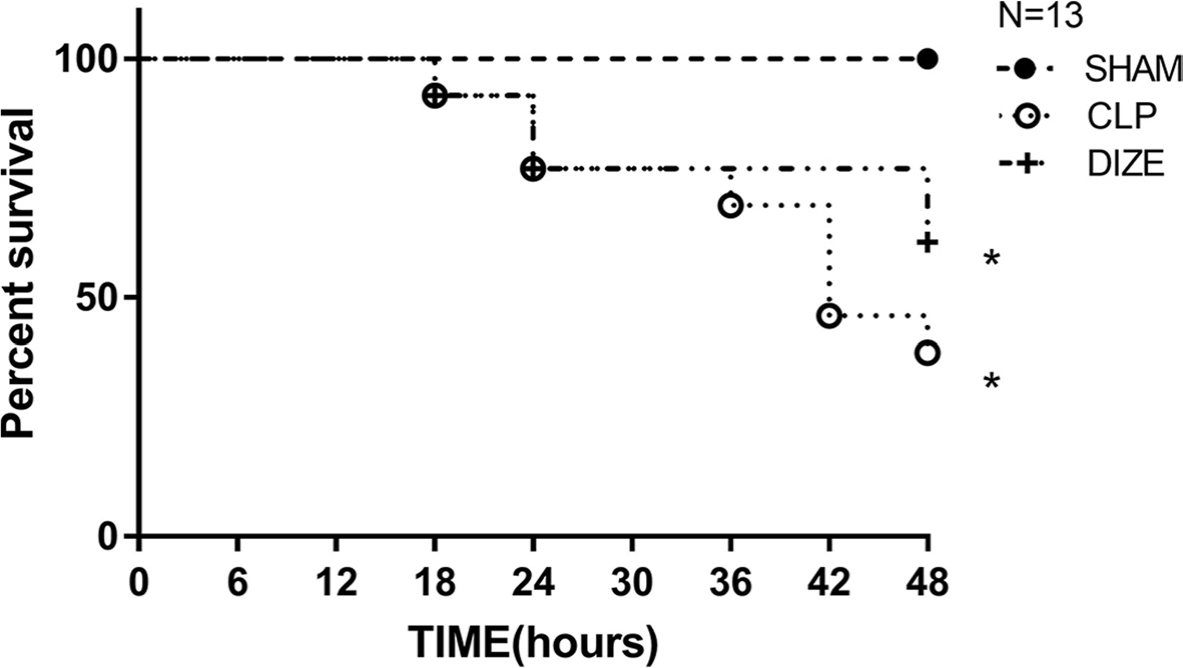
Comparison of overall survival among sham, CLP, and CLP + DIZE treatment groups (*n* = 13 rats/group). * *P* < 0.05 vs sham group. Abbreviations: CLP, cecal ligation and puncture; DIZE, diminazene aceturate

There was a significant difference in mortality among sham, CLP, and CLP + DIZE groups (*P* = 0.004; Fig. 1). Compared with the CLP group, the CLP + DIZE group had a lower mortality rate (38.5% versus 61.5%) at 48 hours post-surgery, although this was not statistically significant.

### Cardiac functions and myocardial injury

**Fig. 2:**
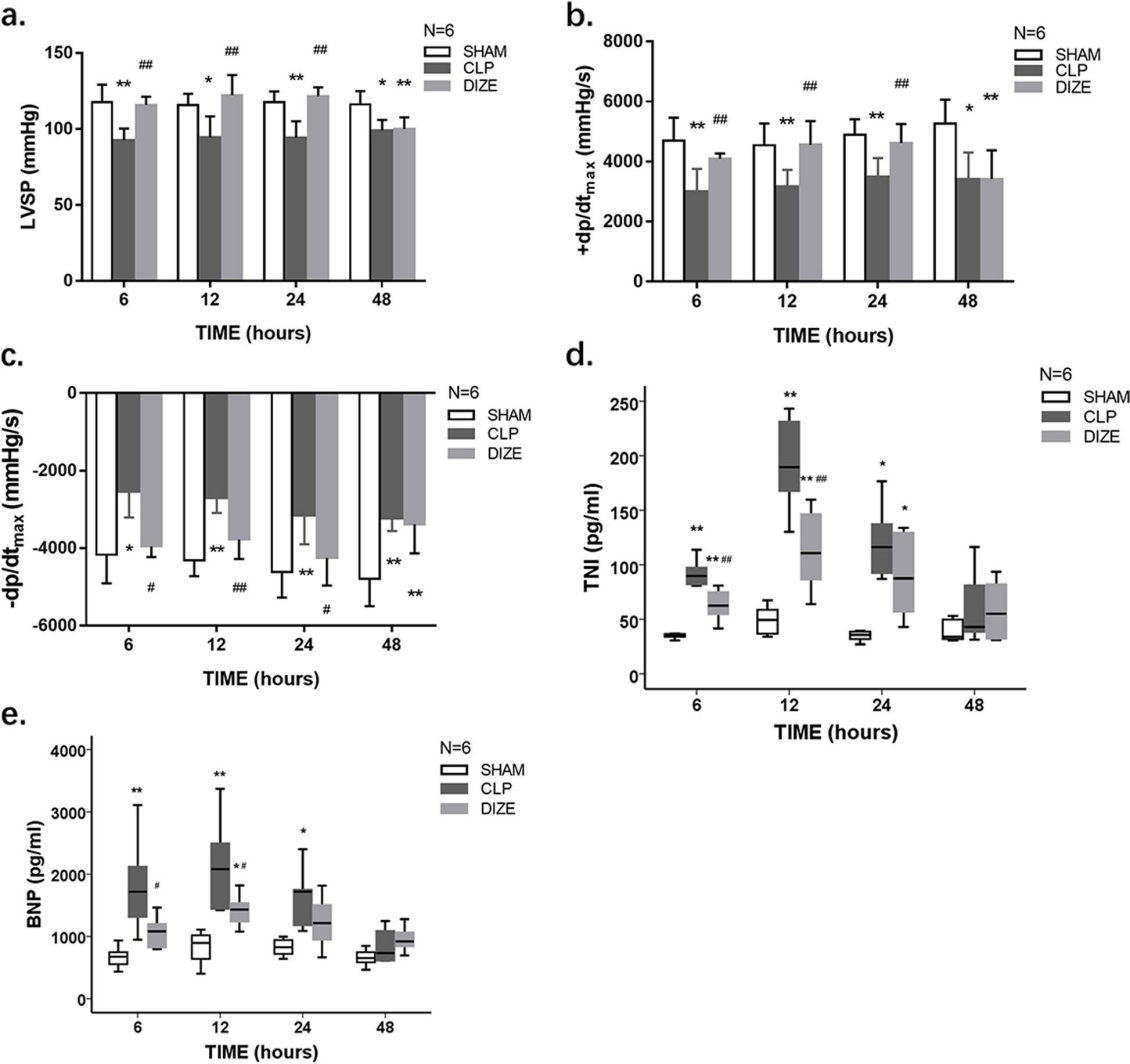
DIZE improved cardiac contractility and reduced plasma BNP and TNI levels. Comparison of LVSP (**a**), +dp/dt_max_ (**b**), and -dp/dt_max_ (**c**), as well as plasma concentrations of TnI (**d**) and BNP (**e**) in the sham, CLP, and CLP + DIZE treatment groups (*n* = 6 rats/group). * *P* < 0.05 vs sham group, ** *P* < 0.01 vs sham group; # *P* < 0.05 vs CLP group, ## *P* < 0.01 vs CLP group. Abbreviations: CLP, cecal ligation and puncture; DIZE, diminazene aceturate; LVSP, left ventricular systolic pressure; +dp/dtmax, maximal left ventricular pressure rising rate; −dp/dt_max_, maximal left ventricular pressure declining rate; TnI, troponin I; BNP, brain natriuretic peptide

Cardiac function and plasma biomarkers that indicated myocardial injury are shown in Fig. 2. The LVSP, +dp/dt_max_, and -dp/dt_max_ in the CLP group were remarkably lower than those in the sham group at 6, 12, 24, and 48 hours after the surgery (LVSP *P* = 0.006, 0.013, < 0.001, and 0.002, respectively; +dp/dt_max_ *P* < 0.001, *P* = 0.007, 0.001, and 0.010, respectively; −dp/dt_max_ *P* = 0.011, < 0.001, 0.003, and 0.001, respectively). Compared with rats in the CLP group, rats in the CLP + DIZE group had significantly improved LVSP, +dp/dt_max_, and -dp/dt_max_ at 6, 12, and 24 hours (LVSP *P* = 0.017, 0.002, and < 0.001, respectively; +dp/dt_max_ *P* = 0.008, 0.005, and 0.005, respectively; −dp/dt_max_ *P* = 0.028, 0.001, and 0.016, respectively). The CLP + DIZE group and the CLP group had no obvious differences in the measurements of LVSP, +dp/dt_max_, or -dp/dt_max_ at 48 hours. Compared with the sham group, the CLP group had significantly higher levels of plasma TnI and BNP at 6, 12, and 24 hours (TNI: *P* < 0.001, < 0.001, and *P* = 0.002, respectively; BNP: *P* = 0.001, < 0.001, and 0.002, respectively). The plasma TnI levels in the CLP + DIZE group were significantly higher than those in the sham group at 6, 12, and 24 hours (*P* < 0.001, < 0.001, and *P* = 0.045, respectively), but were lower than those in the CLP group at 6 and 12 hours (*P* = 0.001 and 0.004, respectively). Meanwhile, the CLP + DIZE group had significantly decreased plasma BNP levels when compared with the CLP group at 6 and 12 hours (*P* = 0.014 and 0.018, respectively).

### Plasma cytokine levels

**Fig. 3:**
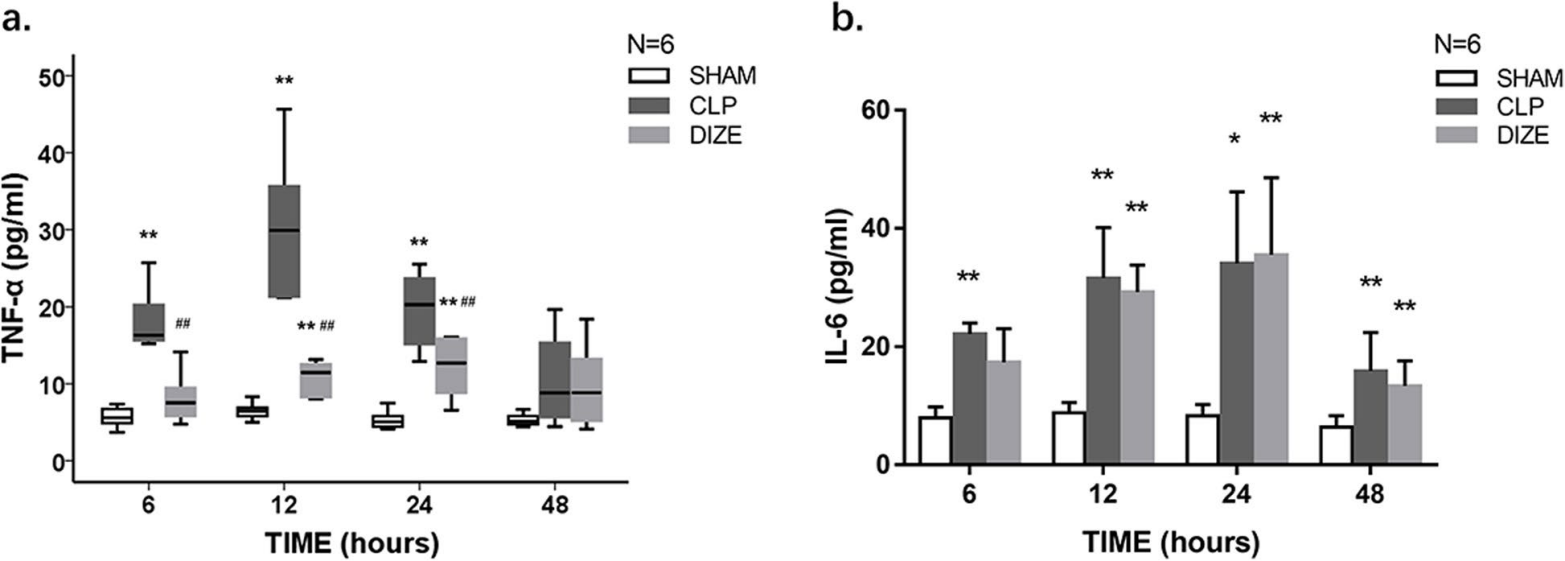
Comparison of plasma TNF-α (**a**) and IL-6 (**b**) levels among sham, CLP, and CLP + DIZE treatment groups (*n* = 6 rats/group). * *P* < 0.05 vs sham group, ** *P* < 0.01 vs sham group; # *P* < 0.05 vs CLP group, ## *P* < 0.01 vs CLP group. Abbreviations: CLP, cecal ligation and puncture; DIZE, diminazene aceturate; TNF-α, tumor necrosis factor-alpha; IL-6, interleukin-6

Comparisons of plasma cytokine levels are shown in Fig. 3. Plasma levels of TNF-α in the CLP group were significantly higher compared to the sham group at 6, 12, and 24 hours (*P* < 0.001, < 0.001, and < 0.001, respectively). At the same timepoints, plasma TNF-α levels in the CLP + DIZE group were significantly lower than those in the CLP group (*P* < 0.001, < 0.001, and *P* = 0.008, respectively). At 6, 12, 24, and 48 hours, the plasma levels of IL-6 in the CLP group were significantly higher compared to the sham group (*P* = 0.002, < 0.001, = 0.012, and = 0.003 respectively). However, there was no significant difference in the plasma IL-6 levels between the CLP + DIZE group and the CLP group at each time point.

### Plasma AngII and Ang-(1–7) levels

**Fig. 4:**
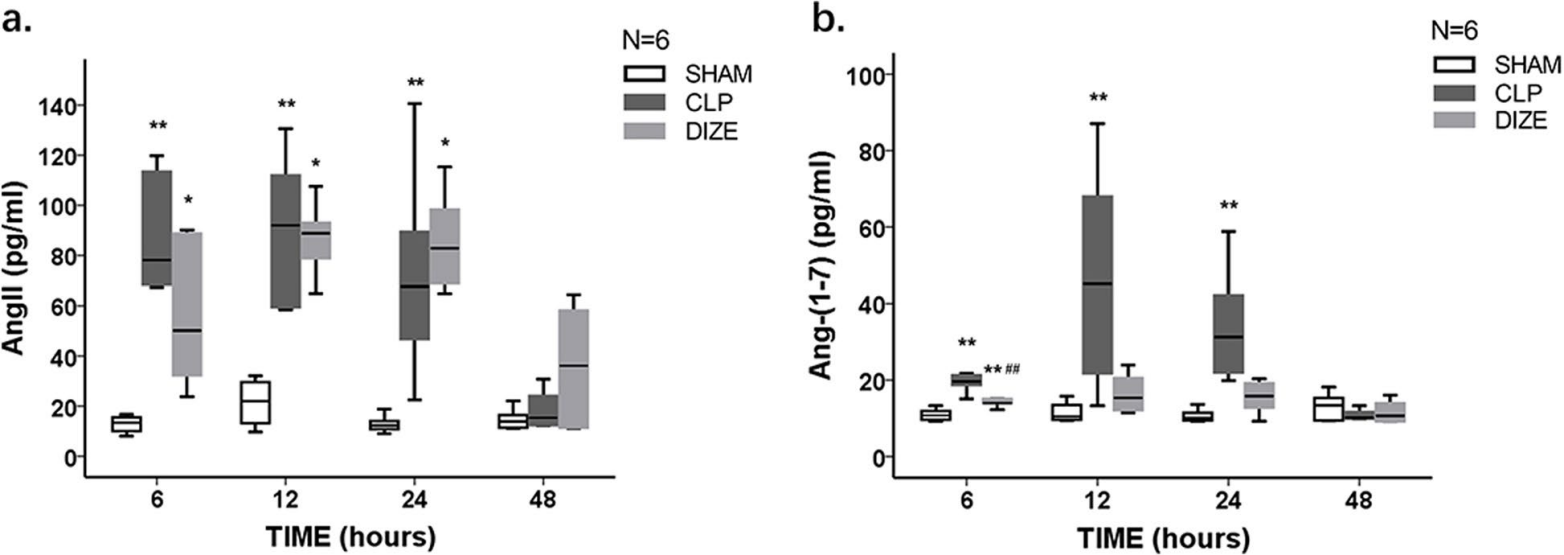
Comparison of plasma AngII (**a**) and Ang-(1–7) (**b**) levels among sham, CLP, and CLP + DIZE treatment groups (*n* = 6 rats/group).* *P* < 0.05 vs sham group, ** *P* < 0.01 vs sham group; ## *P* < 0.01 vs CLP group. Abbreviations: CLP, cecal ligation and puncture; DIZE, diminazene aceturate; AngII, angiotensin II; Ang-(1–7), angiotensin-(1–7)

Plasma AngII levels in the CLP group and CLP + DIZE group were significantly higher than those in the sham group at 6, 12, and 24 hours (CLP group vs. sham group *P* = 0.002, 0.007, and 0.021, respectively; CLP + DIZE group vs. sham group *P* = 0.045, 0.015, and 0.005, respectively; Fig. 4). There was no significant difference in the plasma AngII levels between the CLP + DIZE and CLP groups at each time point.

The plasma Ang-(1–7) levels were significantly higher in the CLP group than in the sham group at 6, 12, and 24 hours (*P* < 0.001, *P* = 0.005, and = 0.001, respectively; Fig. 4). Compared with the CLP group, the CLP + DIZE group showed a significant lower plasma Ang-(1–7) level only at 6 hours post-surgery (*P* = 0.005).

### Myocardial Ang-(1–7) levels

**Fig. 5:**
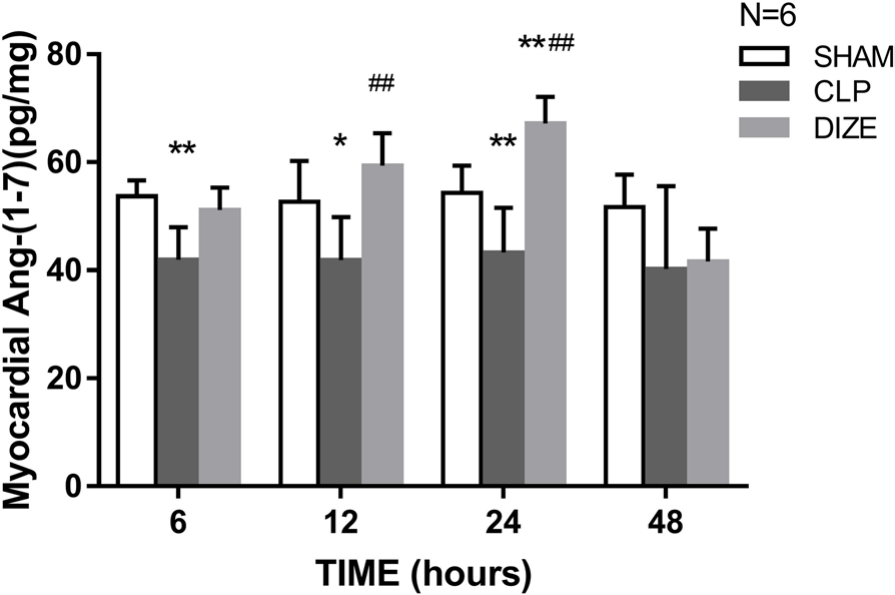
Comparison of myocardial Ang-(1–7) levels among sham, CLP, and CLP + DIZE treatment groups (*n* = 6 rats/group). * *P* < 0.05 vs sham group, ** *P* < 0.01 vs sham group; ## *P* < 0.01 vs CLP group. Abbreviations: CLP, cecal ligation and puncture; DIZE, diminazene aceturate; Ang-(1–7), angiotensin-(1–7)

There was a significant decrease in myocardial Ang-(1–7) levels in the CLP groups compared to the sham groups between 6 and 24 hours post-surgery (*P* = 0.004, *P* = 0.020, and 0.008, respectively; Fig. 5), and this decrease in myocardial Ang-(1–7) levels could be improved by DIZE administration, especially at 12 hours and 24 hours post-operation (*P* = 0.001 and *P* < 0.001, respectively).

### Myocardial ACE, ACE2, AT1R and MasR expressions

**Fig. 6:**
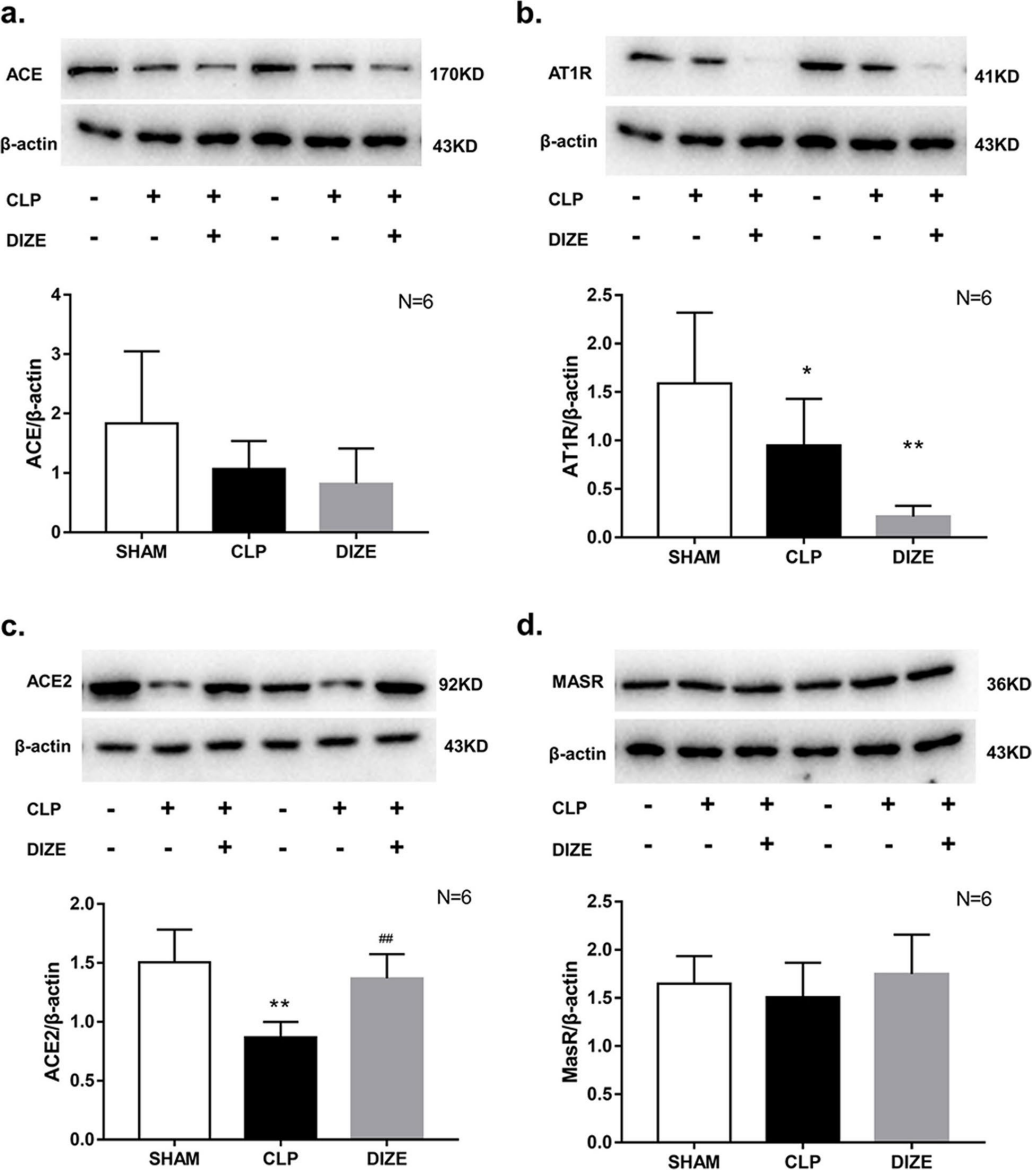
Comparison of myocardial ACE (**a**), AT1R (**b**), ACE2 (**c**), and MasR (**d**) expressions among sham, CLP, and CLP + DIZE treatment groups (*n* = 6 rats/group). * *P* < 0.05 vs sham group; ** *P* < 0.01 vs sham group; # *P* < 0.05 vs CLP group. Abbreviations: CLP, cecal ligation and puncture; DIZE, diminazene aceturate; ACE, angiotensin-converting enzyme; AT1R, angiotensin type 1 receptor; ACE2, angiotensin-converting enzyme 2; MasR, Mas receptor

The expression of AT1R and ACE2 in the myocardium of the CLP group were significantly decreased compared to the sham group at 24 hours post-surgery (Fig. 6; AT1R *P* = 0.047; ACE2 *P* < 0.001). However, there were no differences between the sham and CLP group in the expression of ACE and MasR. The level of ACE2 was significantly increased in the CLP + DIZE group compared to the CLP group (*P* < 0.001). The AT1R level was significantly decreased in the CLP + DIZE group compared to the sham group (*P* = 0.002), but was not different from the CLP group.

### Histological examinations

**Fig. 7:**
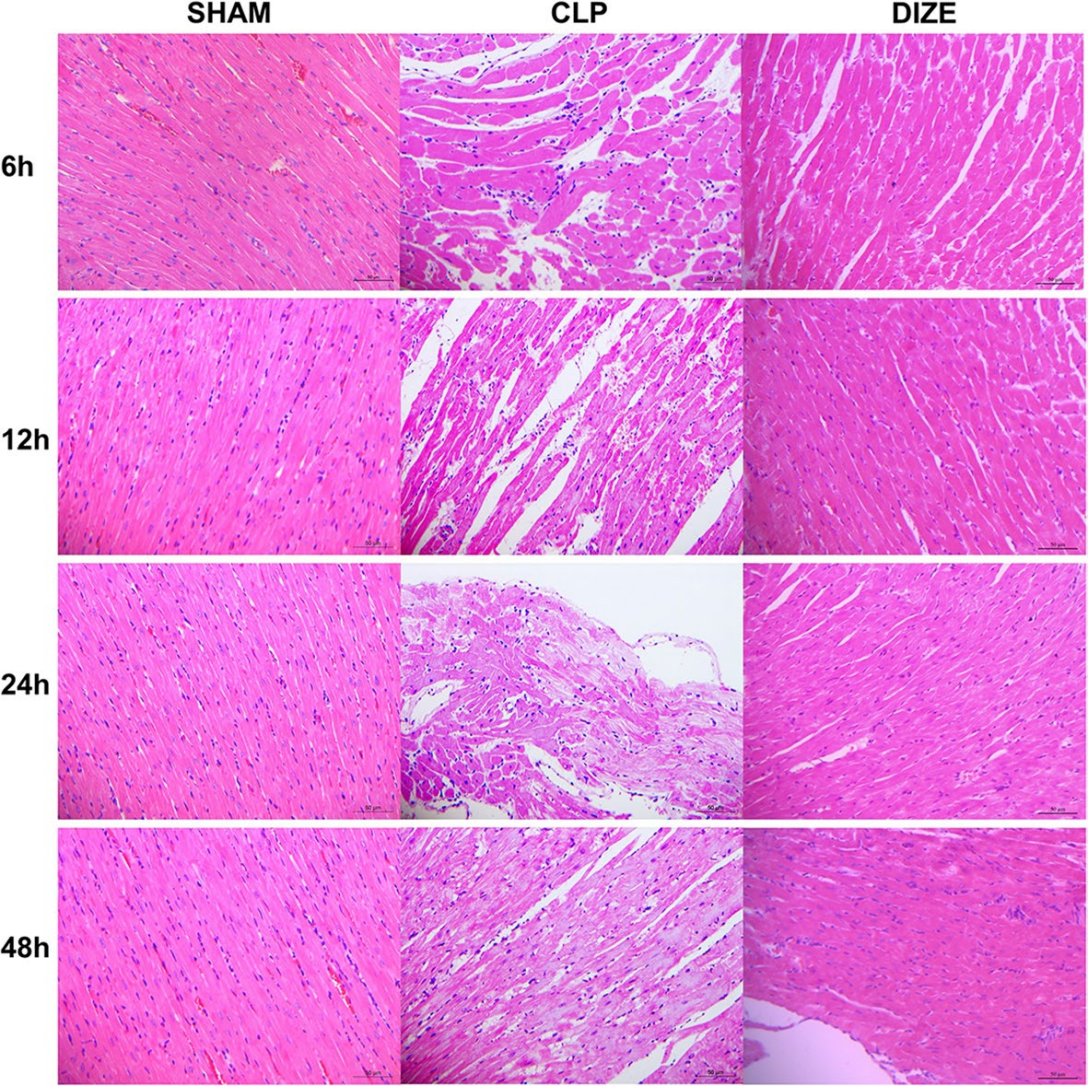
DIZE minimized histological disruptions of myocardial tissues. Representative pathological changes of myocardial tissues at four different time points (6, 12, 24 and 48 h) post-surgery from sham, CLP, and CLP + DIZE-treated rats (hematoxylin and eosin staining, magnification × 400). Abbreviations: CLP, cecal ligation and puncture; DIZE, diminazene aceturate

Vacuolar degeneration of myocardial cells, along with few local inflammatory cell infiltration, was observed in tissues from the CLP group at 6 hours post-surgery (Fig. 7). These changes were exaggerated at 12 and 24 hours after the operation when myocyte lysis started to appear. These degenerations were improved at 48 hours post-surgery, although the disorganized structure of the tissue persisted. The CLP + DIZE group had less severe myocardial injuries compared with the CLP group at each timepoint, especially at 12 and 24 hours post-surgery.

**Fig. 8:**
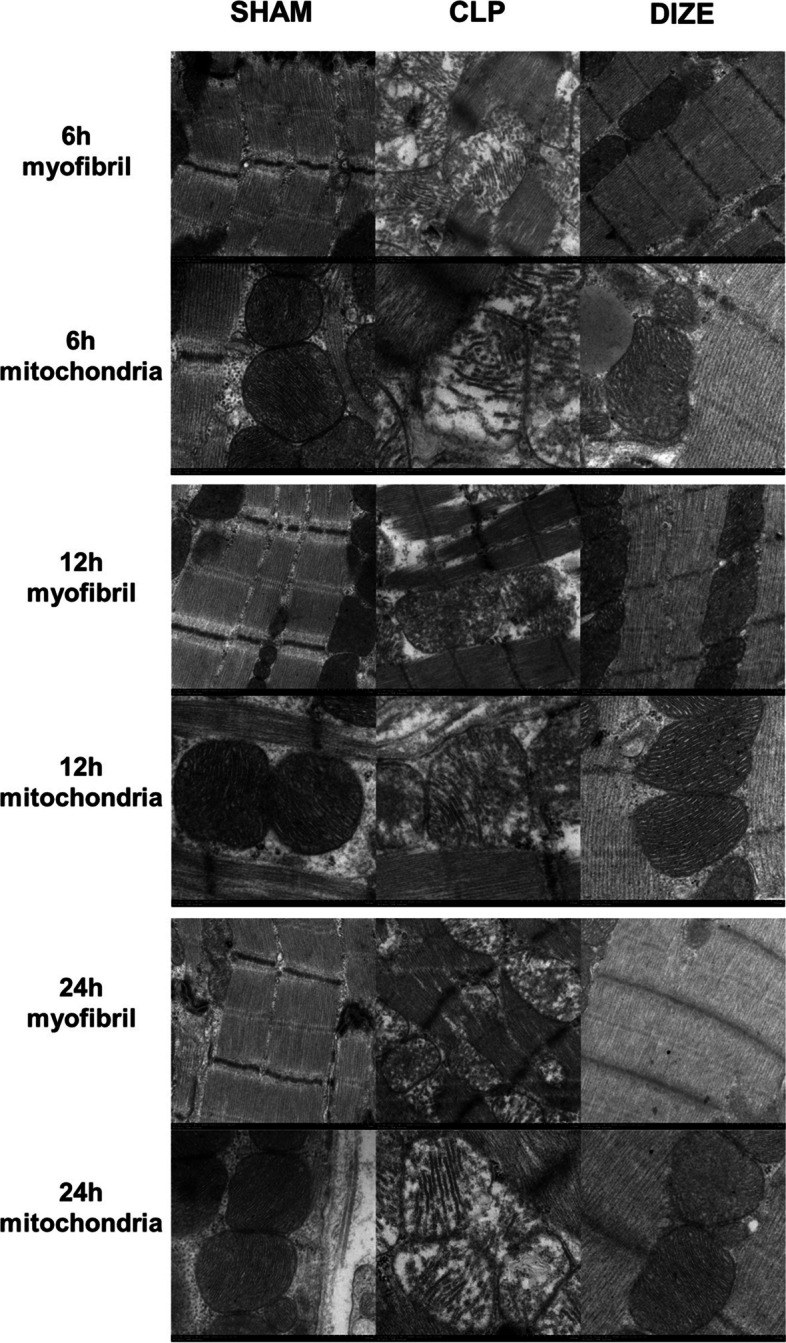
DIZE improved myocardial ultrastructural damages showed by TEM. Representative TEM images of myocardial tissues at three times points (6, 12, and 24 h) post-surgery from sham, CLP, and CLP + DIZE-treated rats (pictures for myofibril, magnification × 50,000; pictures for mitochondria, magnification × 100,000). Abbreviations: TEM, transmission electron microscopy; CLP, cecal ligation and puncture; DIZE, diminazene aceturate

Transmission electron microscopy showed that the rats in the CLP group had mitochondrial swelling, decreased electron density, mitochondrial membrane destruction, cristae reduction, and myofibril dissolution at each time point (Fig. 8). Compared to the CLP group, the CLP + DIZE group showed significantly fewer injuries to the mitochondria and myofilaments at each time point between 6 and 24 hours. This protective effect of DIZE on the cellular microstructures was easily observed in the mitochondria.

## Discussion

Cardiovascular dysfunction is a common complication of sepsis [3]. Septic patients with cardiac symptoms have high mortality rates [5]. Despite a lack of uniform diagnostic criteria, SCM is accepted as an acute syndrome of sepsis-associated myocardial dysfunction that is not due to cardiac ischemia and carries some of the following features: (1) dilated left ventricle with normal- or low-filling pressure; (2) impaired contractility in ventricles; and (3) right or left ventricular (systolic or diastolic) dysfunction with a decreased response to volume expansion [4, 25]. Importantly, it is believed that RAS is involved in the development of cardiovascular disorders [8]. In the current study, a rat CLP model of sepsis was used to study the effects of DIZE in the development of SCM. For the past 60 years, DIZE has been used to treat protozoan and babesia infections in animals [10], as well as trypanosomiasis in humans [11]. In 2008, Hernández and colleagues found that DIZE can activate ACE2 in vitro [26]. Therefore, DIZE has since been used in cardiovascular research. Studies have shown DIZE to have an antihypertensive effect, similar to that of ACE inhibitors [27, 28]. In models of acute myocardial infarction and chronic heart failure, the administration of DIZE could reduce the size of the myocardial infarction and improve ventricular systolic and diastolic function, as well as inhibit myocardial fibrosis [14, 20, 24, 29–31]. The underlying protective mechanism of DIZE on the cardiovascular system is still unclear.

Qi et al. administered DIZE intraperitoneally before and after an operation in a rat model of acute myocardial infarction [14]. After four weeks, DIZE treatment decreased the infarction area and improved the ventricular contractility by increasing the activity of plasma and myocardial ACE2, raising myocardial ACE2 and MasR mRNA levels, and depressing myocardial expression of ACE and AT1R mRNA compared to the myocardial infarction group. It was concluded that DIZE could promote the ACE2/Ang-(1–7)/MasR axis and inhibit the ACE/AngII/AT1R axis to play a protective role in the development of myocardial infarction. The study by Awward et al. had similar results [24]. In this experiment, subcutaneous injections of DIZE (15 mg/kg) for 21 days could mitigate the effects of chronic heart failure caused by pregabalin. This study also revealed increased Ang-(1–7), ACE2, and MasR, and decreased ACE and AT1R expression in the myocardium. Another study reported that the administration of DIZE for four weeks could significantly increase myocardial ACE2 protein concentration after the myocardial infarction, as well as improve cardiac function by preserving carotid flow, stroke volume, and cardiac output [20]. Taken together, these data support the role of DIZE in improving the function of Ang-(1–7)/MasR by activating ACE2 and elevating cardiac ACE2 protein to play a protective role in the pathogenesis of cardiovascular disorders.

The present study evaluated cardiac function, myocardial pathology, plasma cytokine levels, and changes to RAS at different postoperative time points in the rat CLP model of sepsis. The role of DIZE was investigated in the development of SCM in this model of sepsis for the first time. The results demonstrated that the administration of DIZE could significantly reduce plasma levels of the pro-inflammatory factor TNF-α in septic rats, and improve myocardial contractility (±dp/dt_max_) and LVSP, which was parallel with reduced plasma levels of the cardiac function marker BNP. Furthermore, consistent with the amelioration of cardiac function and the decreased levels of myocardial injury biomarker TnI, the histological examination revealed that DIZE could significantly reduce the loosening and breakage of myocardial fibers and improve myocardial mitochondrial damage, which in turn mitigates the development of SCM and reduces sepsis-associated mortality. The effect of DIZE was correlated with increased myocardial ACE2 and Ang-(1–7) levels, as well as decreased AT1 expression, although it had little correlation with the changes in plasma AngII and Ang-(1–7) levels. In the myocardium, DIZE could prevent the ACE2 expression decrease induced by sepsis, which restored the local Ang-(1–7) levels to protect the myocardium and improved the ventricular systolic function that finally led to decreased plasma levels of TnI and BNP. The human DIZE pharmacokinetics have not been reported. In animals, the half-life of DIZE was found to be 11–14 hours for sheep, dogs and goats, and 63 hours for cattle [11]. Therefore, the decreased myocardial protective effect of DIZE at 48 hours might be due to its clearance from the rats.

The main pathological mechanisms of SCM include dysregulated inflammatory mediators, oxidative stress, mitochondrial dysfunction, calcium regulation disorders, autonomic nervous system disorders, and dysfunction of the endothelial barrier [4, 5]. Kuriakose et al. revealed that, in the macrophages activated by endotoxin, DIZE could reduce the nuclear factor kappa-B (NF-κB) activity by down-regulating the phosphorylation of mitogen-activated protein kinase (MAPK)/extracellular signal related kinase (ERK) and signal transducer and activator of transcription (STAT), which in turn decreased the expression of pro-inflammatory factors, such as TNF-α and IL-6 [32]. Another study conducted by Collins et al. showed that Ang-(1–7) could affect RAW 264.7 cells by inhibiting TNF-α production in a dose-dependent manner [33]. These results were supported by several other studies [34–37], and were consistent with the decreased plasma TNF-α levels and increased myocardial Ang-(1–7) concentrations in septic rats after DIZE treatment observed in the current study. Therefore, it was speculated that DIZE could reduce the local myocardial damage caused by inflammatory factors through the ACE2/Ang-(1–7)/Mas axis.

Studies regarding the role of the RAS system in sepsis have also shown that Ang-(1–7) could attenuate the AngII-stimulated enhancement in lipid peroxidation and reduce the superoxide dismutase activity in murine hearts [38]. It was previously reported that Ang-(1–7) could stimulate protein kinase A (PKA) production via the activation of MasR, which was followed by an enhanced calcium current in the heart. This mechanism thought to regulate calcium homeostasis and improve heart contractility [39]. Furthermore, Xu et al. reported that administration of Ang-(1–7) could reverse the LPS-induced reduction of α-myosin heavy chain (MHC) and β-MHC, and increase levels of S100 calcium binding protein A8 (S100A8) and S100A9 in the mouse left ventricle and finally ameliorate ventricular contractility [34]. In summary, in addition to reducing the expression of pro-inflammatory factors, Ang-(1–7) also reduced the generation of myocardial oxygen free radicals, increased the influx of calcium ions in myocardial cells, and elevated the expressions of myosin and S100 calcium binding proteins, thereby enhancing myocardial contraction, maintaining calcium homeostasis in the myocardium, and protecting mitochondria.

Our experiments showed that DIZE increased cardiac ACE2 expression, and augmented myocardial Ang-(1–7) production, which could result in reduced expression of pro-inflammatory factors, decreased myocardial oxidative stress, increased myocardial calcium influx, increased myosin and S100 calcium binding protein expression, and minimize mitochondrial damage through the MasR pathway. The outcomes were preserved intact structures and functions of mitochondria, maintained calcium homeostasis, and reduced myocardial damage to improve myocardial activities and mitigate the development of SCM.

In addition, it has been established that silencing AT1R plays an inhibitory role in the SCM by blocking the MAPK signaling pathway (demonstrated as reduced extracellular signal-regulated kinase and cyclic adenosine 3′,5′-monophosphate response element binding protein expressions, and decreased concentrations of TnI, troponin T, and creatine kinase-MB) [40]. Therefore, DIZE may also mitigate SCM through diminished myocardial AT1R expression by mechanisms other than promoting the ACE2/Ang-(1–7)/MasR pathway.

The current study had several limitations. Firstly, only male rats were used in this study. As such, these results might not be generalized to female rats. Secondly, the observations made models from initially healthy animals might not accurately reflect the clinical situation of patients with sepsis, as patients with sepsis invariably have additional underlying conditions. Thirdly, unlike clinical treatment, antibiotics were not used in this study since antibiotics can affect the expression and activity of various cytokines, which would have introduced confounding factors. Finally, a larger sample size with a longer observation period may be required to show the statistically significant survival benefit of DIZE in the CLP + DIZE group.

## Conclusions

SCM is a serious complication of sepsis without an effective method of treatment. In the present study, it was shown for the first time that DIZE treatment could significantly improve myocardial systolic and diastolic functions, reduce plasma pro-inflammatory factors, such as TNF-α, and myocardial injury biomarkers, such as TnI, and BNP, improve myocardial pathological changes, and reduce mortality. Its effect was related to the promotion of myocardial ACE2 expression and myocardial Ang-(1–7) levels. Future research should focus on the production of myocardial inducible nitric oxide synthase (iNOS), oxygen free radicals, NF-κB, and the inhibitor of NF-κB (IκB) after DIZE treatment to further explore the mechanism of DIZE in mitigating the development of SCM during sepsis.

## Supplementary Information

**Additional file 1.**

**Additional file 2.**

## Data Availability

The datasets used during the current study are available from the corresponding author on reasonable request.

## Abbreviations

SCM: Septic cardiomyopathy
RAS: Renin-angiotensin system
ACE: Angiotensin-converting enzyme
Ang: Angiotensin
AT1R: Angiotensin type 1 receptor
MasR: Mas receptor
DIZE: Diminazene aceturate
CLP: Cecal ligation and puncture
LVSP: Left ventricular systolic pressure
+dp/dtmax: Maximal left ventricular pressure rising rate
-dp/dtmax: Maximal left ventricular pressure declining rate
ELISA: Enzyme-linked immunosorbent assay
TNF-α: Tumor necrosis factor-alpha
IL-6: Interleukin-6
TnI: Troponin I
BNP: Brain natriuretic peptide
ECL: Enhanced chemiluminescence
IQR: Interquartile range
ANOVA: One-way analysis of variance
NF-κB: Nuclear factor kappa-B
MAPK: Mitogen-activated protein kinase
ERK: Extracellular signal related kinase
STAT: Signal transducer and activator of transcription
iNOS: Inducible nitric oxide synthase
IκB: Inhibitor of NF-κB
MHC: Myosin heavy chain
S100A8: Calcium binding protein A8
