# Supplementary material for: Diminazene aceturate mitigates cardiomyopathy by interfering with renin-angiotensin system in a septic rat model
Source: BMC Pharmacol Toxicol. 2022 Jul 4;23:44. doi: 10.1186/s40360-022-00584-4 (PMC9251020; doi:10.1186/s40360-022-00584-4)
Supplement: Supplementary file 2 — Additional file 2. [file 40360_2022_584_MOESM2_ESM.pptx]

## Slide 1
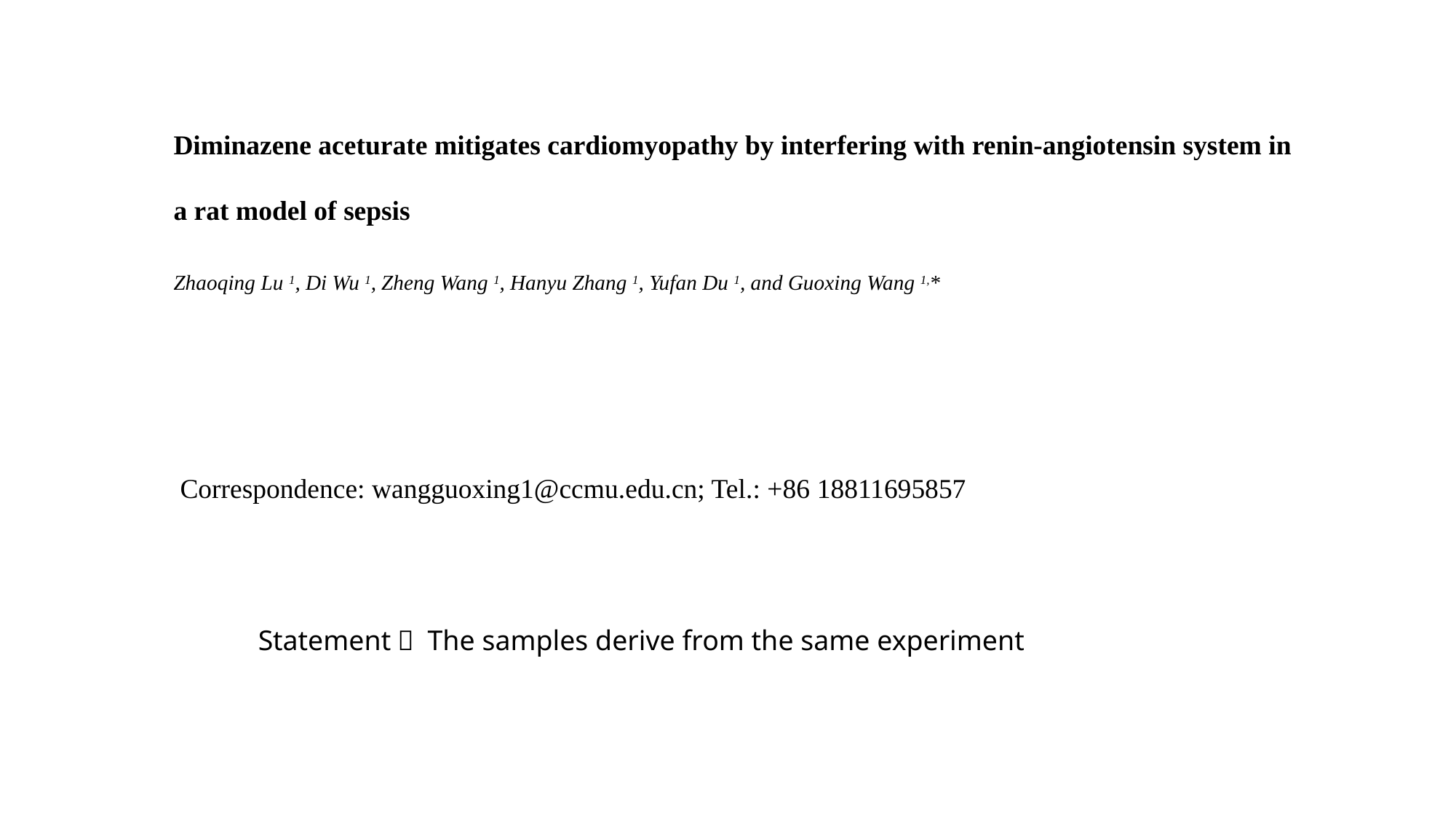

Diminazene aceturate mitigates cardiomyopathy by interfering with renin-angiotensin system in a rat model of sepsis
Zhaoqing Lu 1, Di Wu 1, Zheng Wang 1, Hanyu Zhang 1, Yufan Du 1, and Guoxing Wang 1,*
Correspondence: wangguoxing1@ccmu.edu.cn; Tel.: +86 18811695857
Statement： The samples derive from the same experiment

## Slide 2
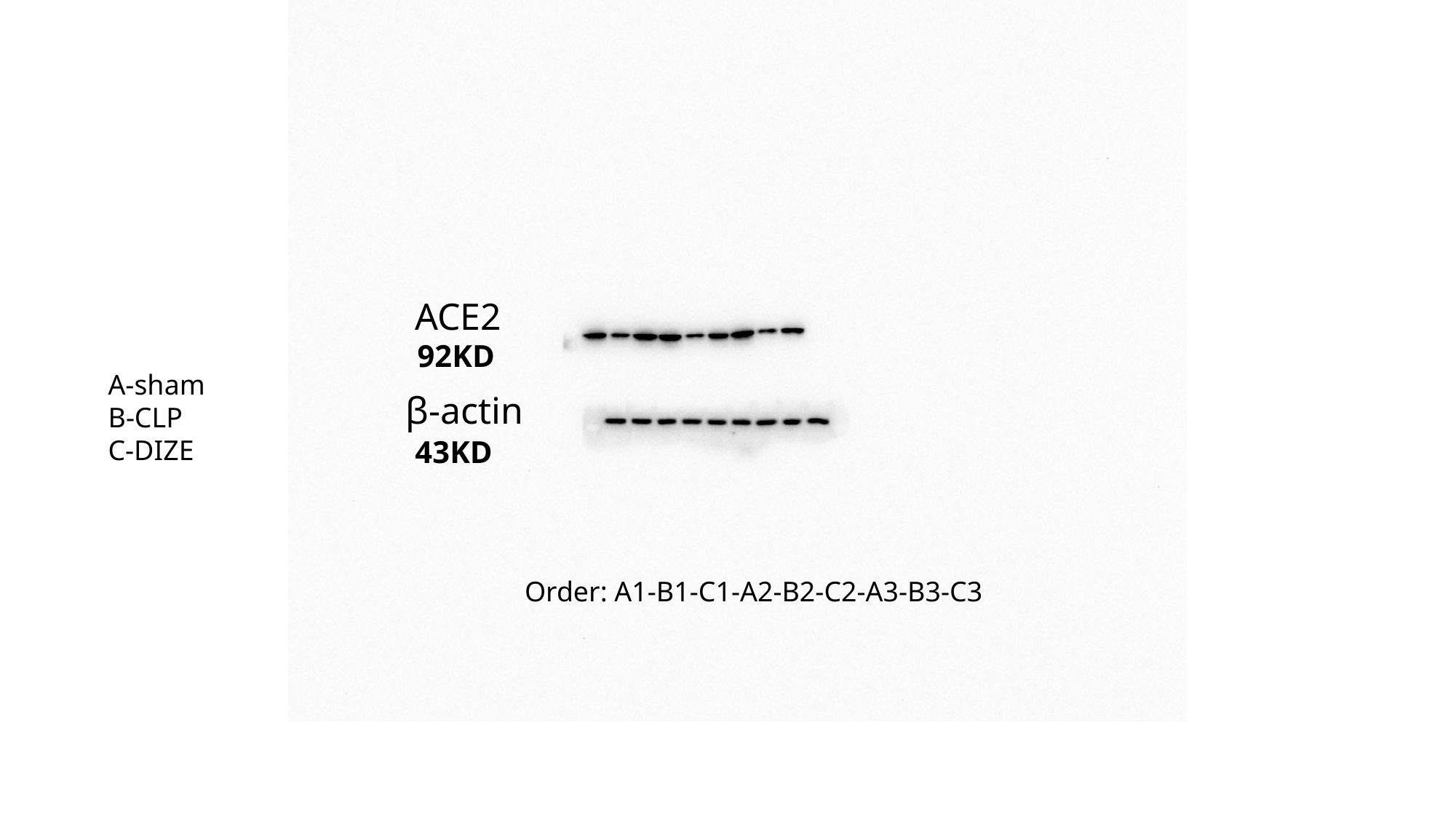

ACE2
92KD
A-sham
B-CLP
C-DIZE
β-actin
43KD
Order: A1-B1-C1-A2-B2-C2-A3-B3-C3

## Slide 3
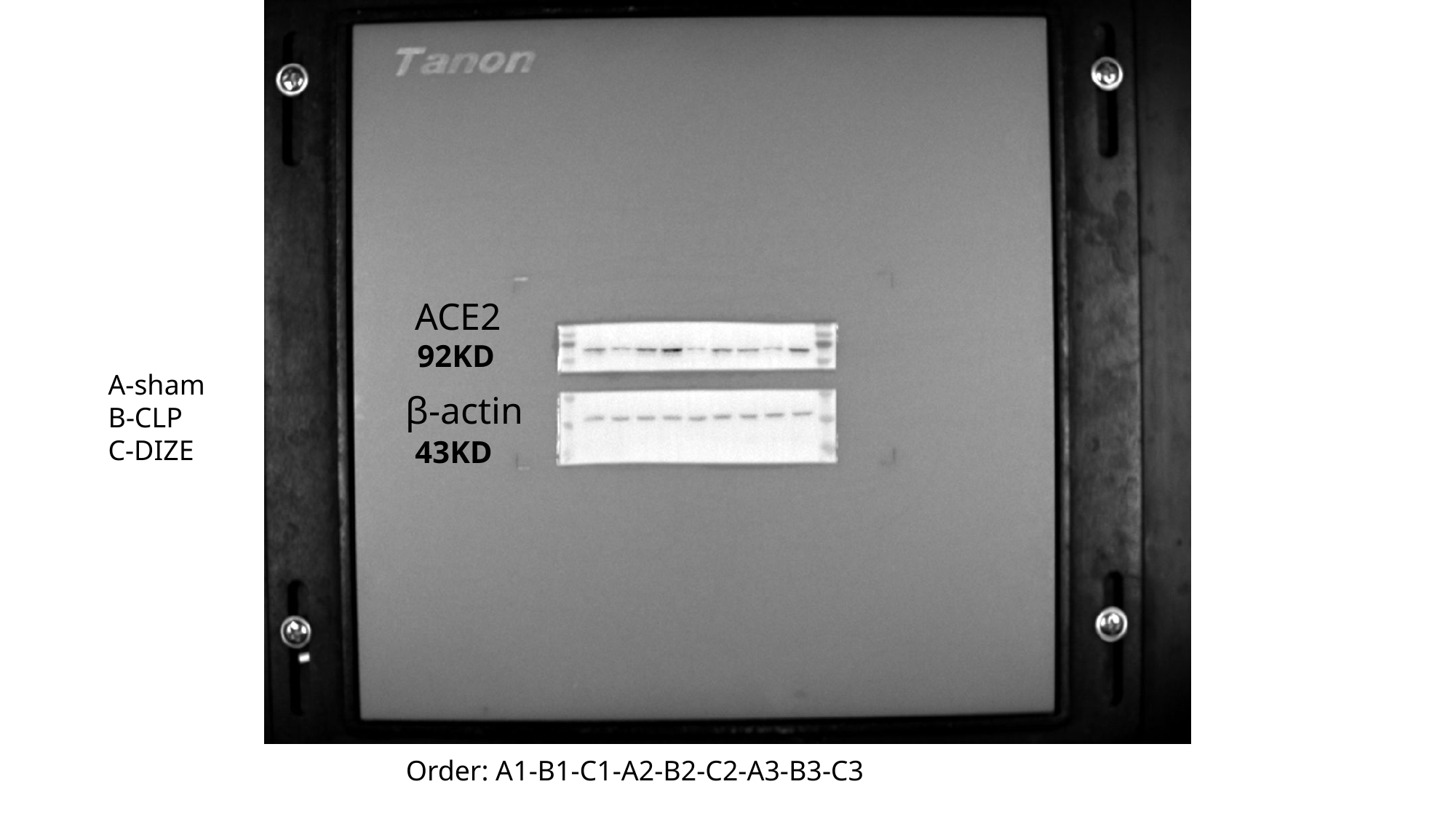

ACE2
92KD
A-sham
B-CLP
C-DIZE
β-actin
43KD
Order: A1-B1-C1-A2-B2-C2-A3-B3-C3

## Slide 4
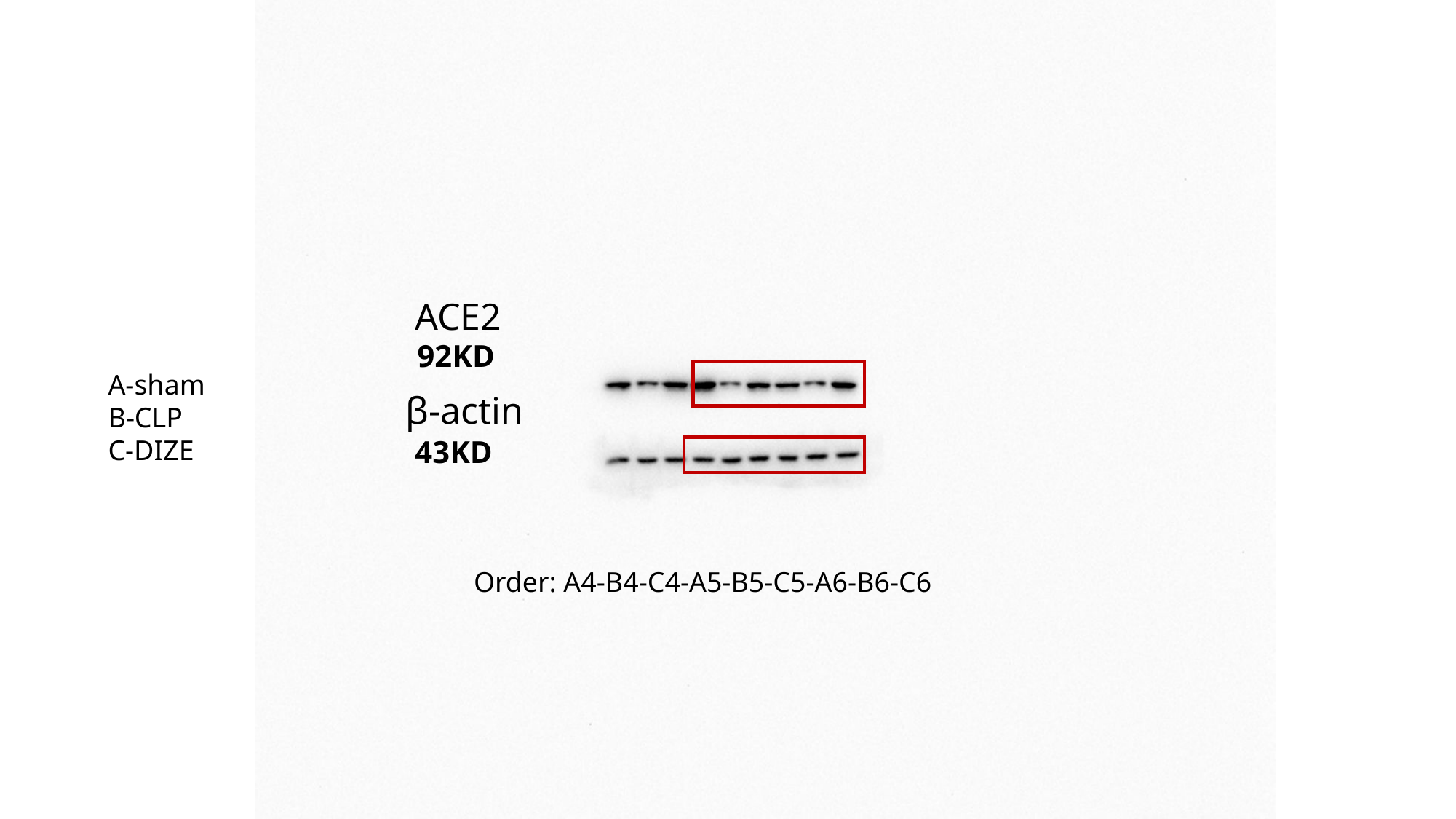

ACE2
92KD
A-sham
B-CLP
C-DIZE
β-actin
43KD
Order: A4-B4-C4-A5-B5-C5-A6-B6-C6

## Slide 5
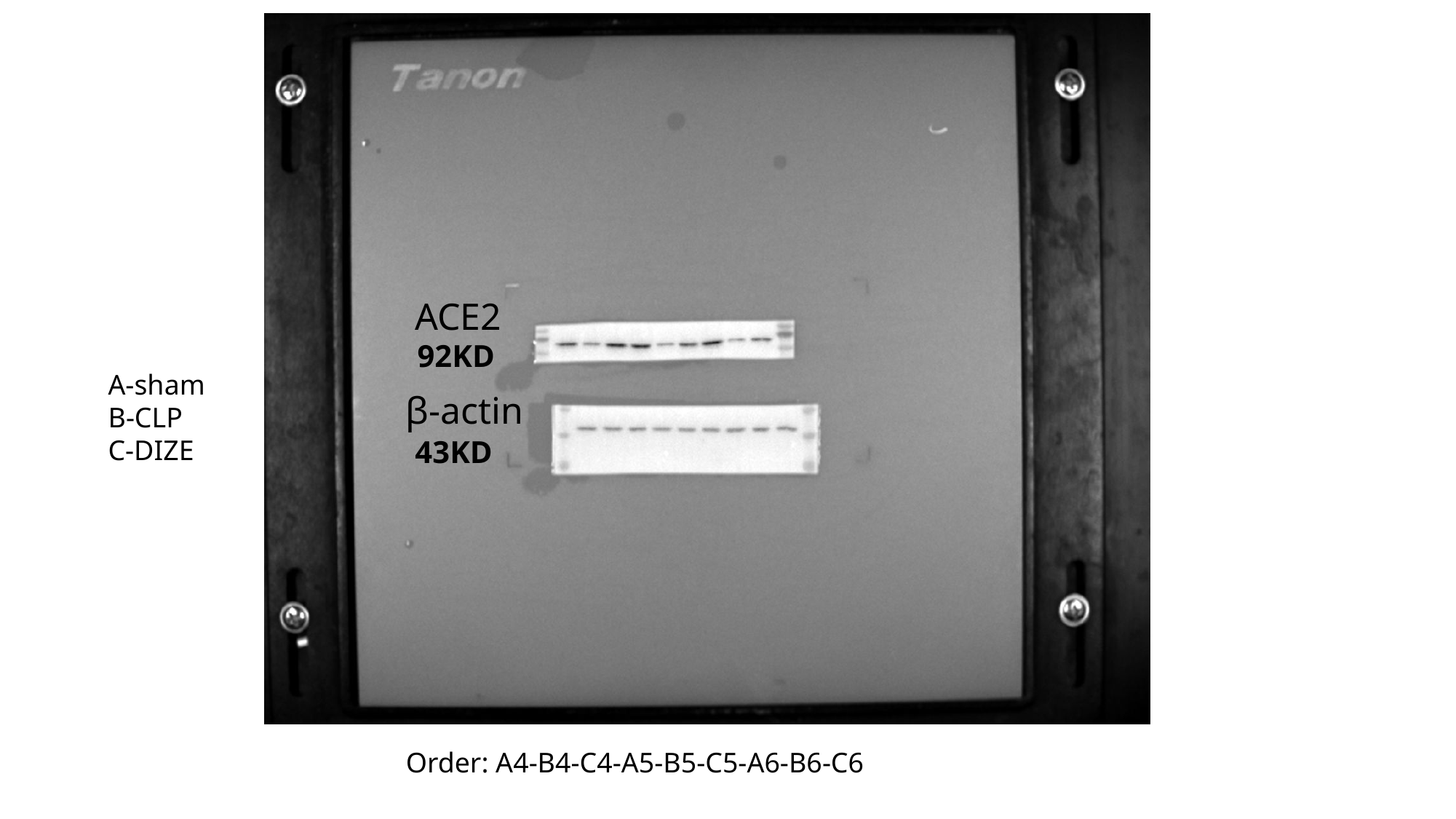

ACE2
92KD
A-sham
B-CLP
C-DIZE
β-actin
43KD
Order: A4-B4-C4-A5-B5-C5-A6-B6-C6

## Slide 6
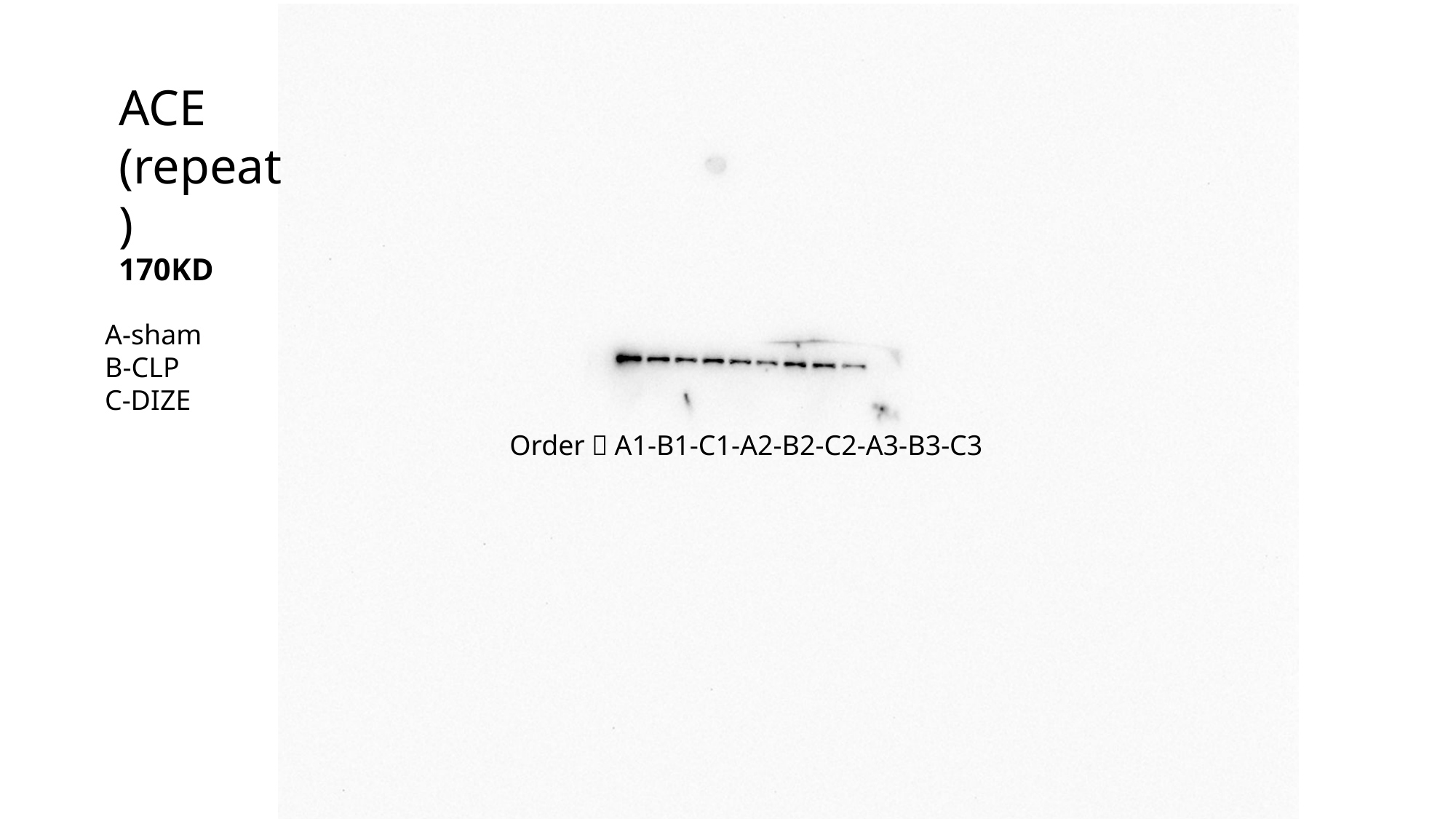

ACE
(repeat)
170KD
A-sham
B-CLP
C-DIZE
Order：A1-B1-C1-A2-B2-C2-A3-B3-C3

## Slide 7
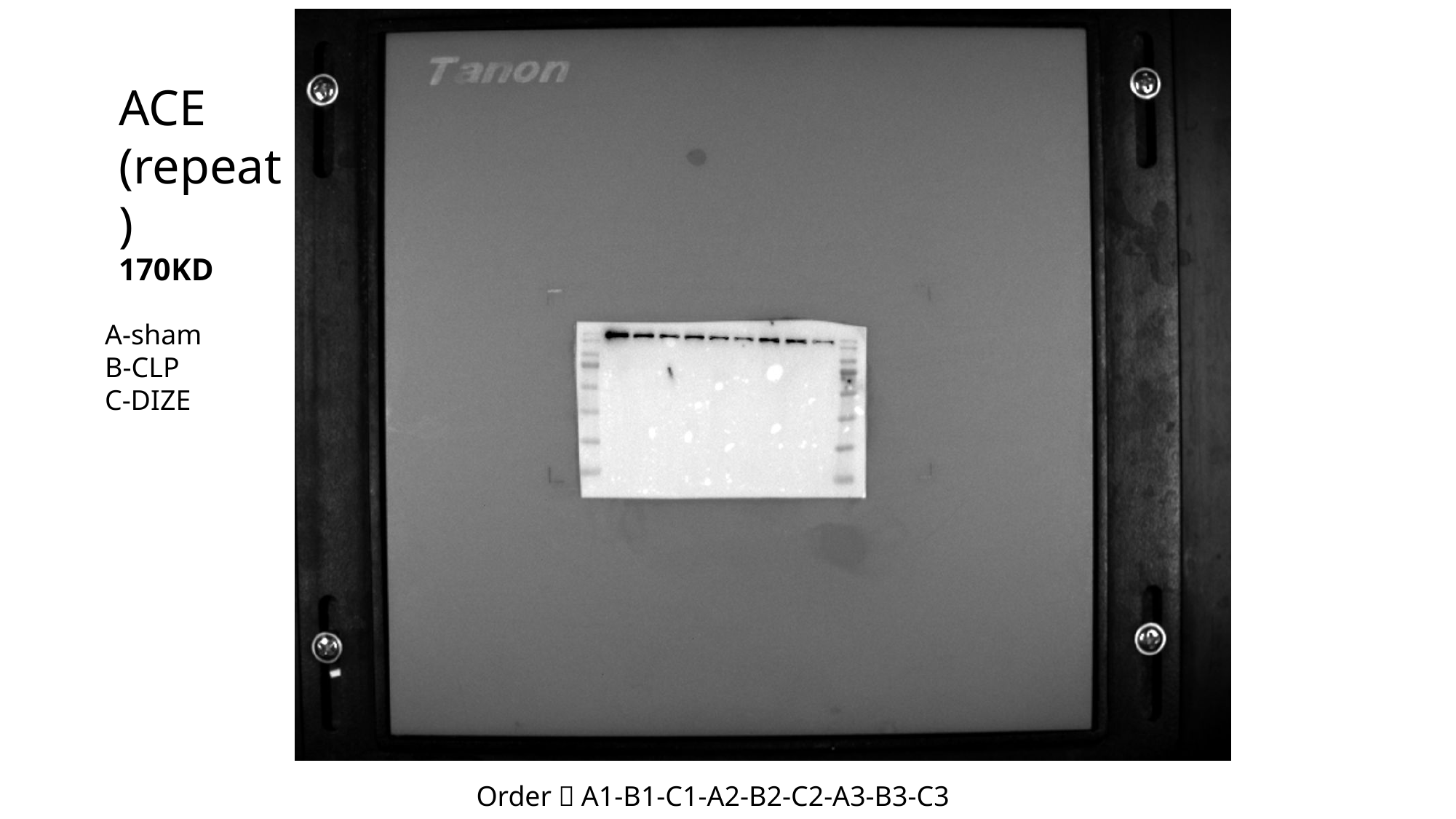

ACE
(repeat)
170KD
A-sham
B-CLP
C-DIZE
Order：A1-B1-C1-A2-B2-C2-A3-B3-C3

## Slide 8
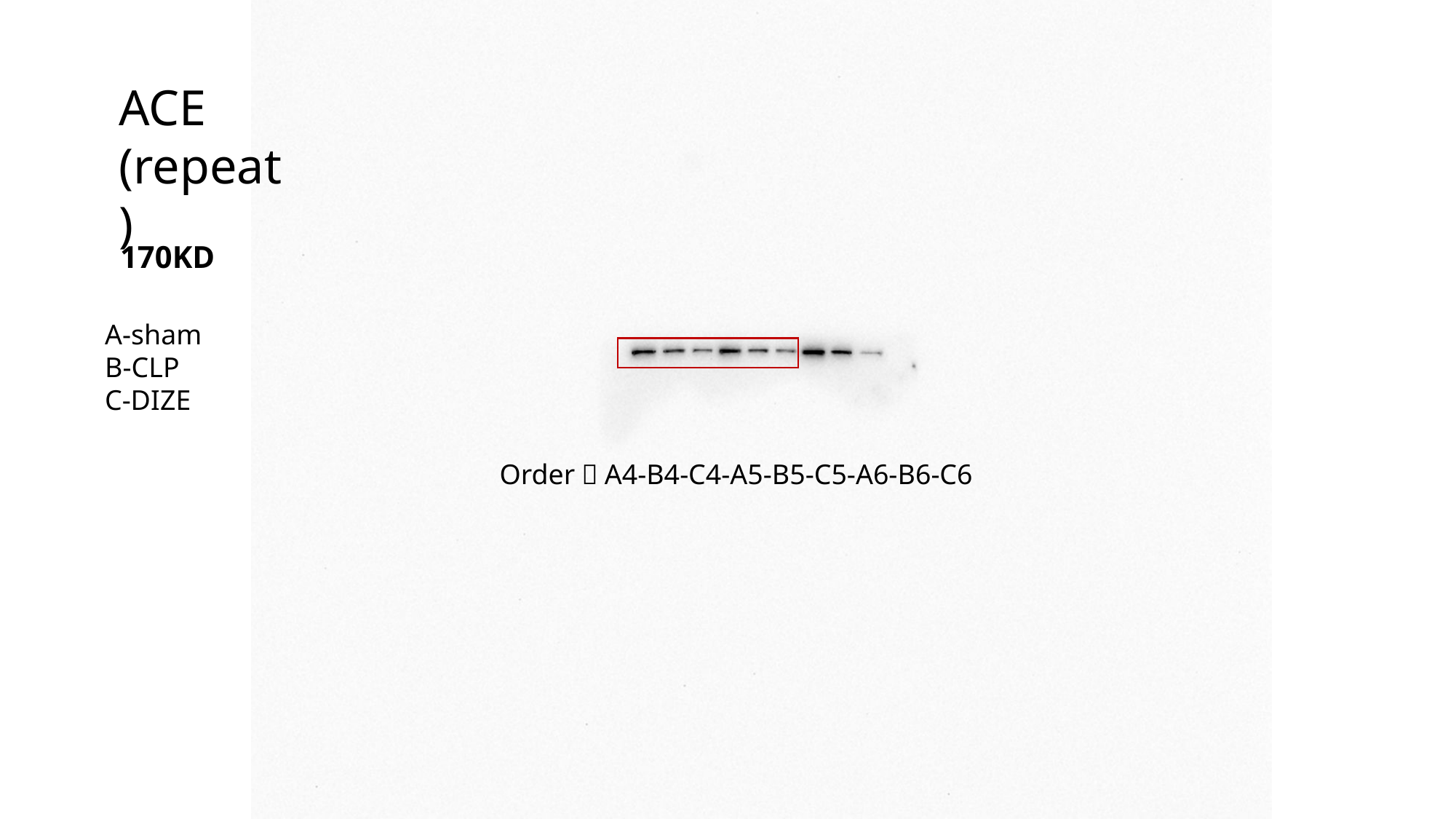

ACE (repeat)
170KD
A-sham
B-CLP
C-DIZE
Order：A4-B4-C4-A5-B5-C5-A6-B6-C6

## Slide 9
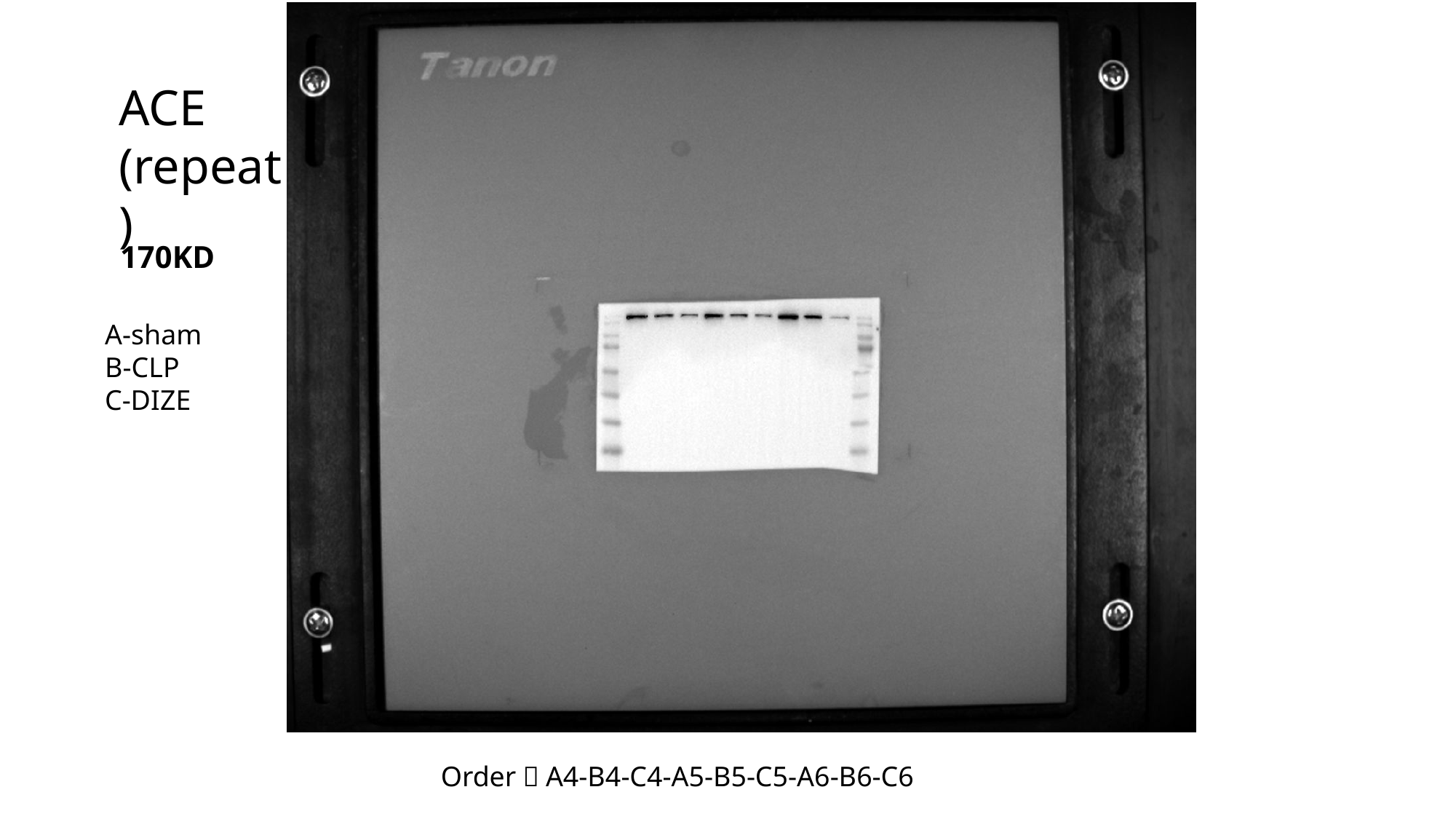

ACE (repeat)
170KD
A-sham
B-CLP
C-DIZE
Order：A4-B4-C4-A5-B5-C5-A6-B6-C6

## Slide 10
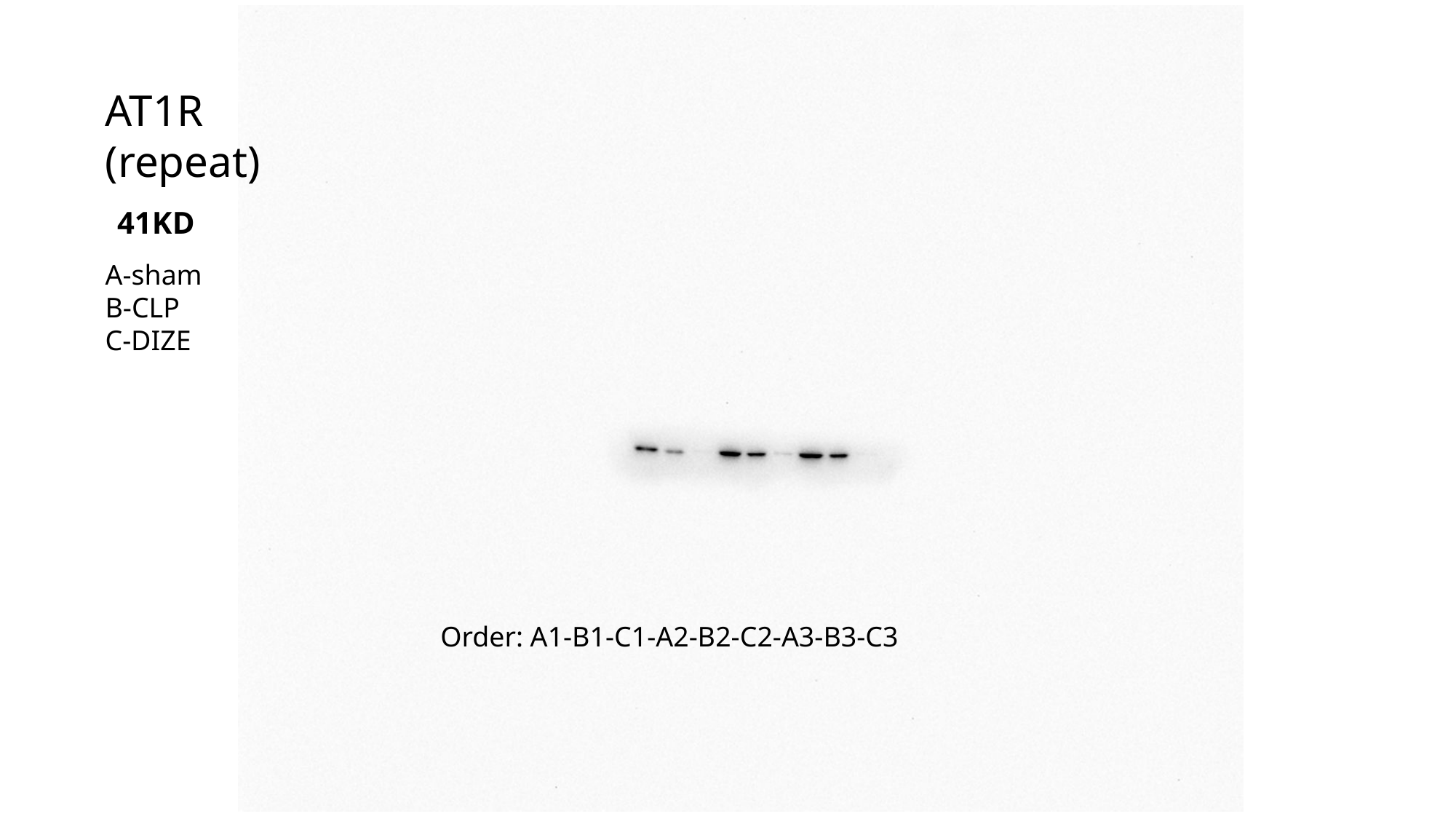

AT1R (repeat)
41KD
A-sham
B-CLP
C-DIZE
Order: A1-B1-C1-A2-B2-C2-A3-B3-C3

## Slide 11
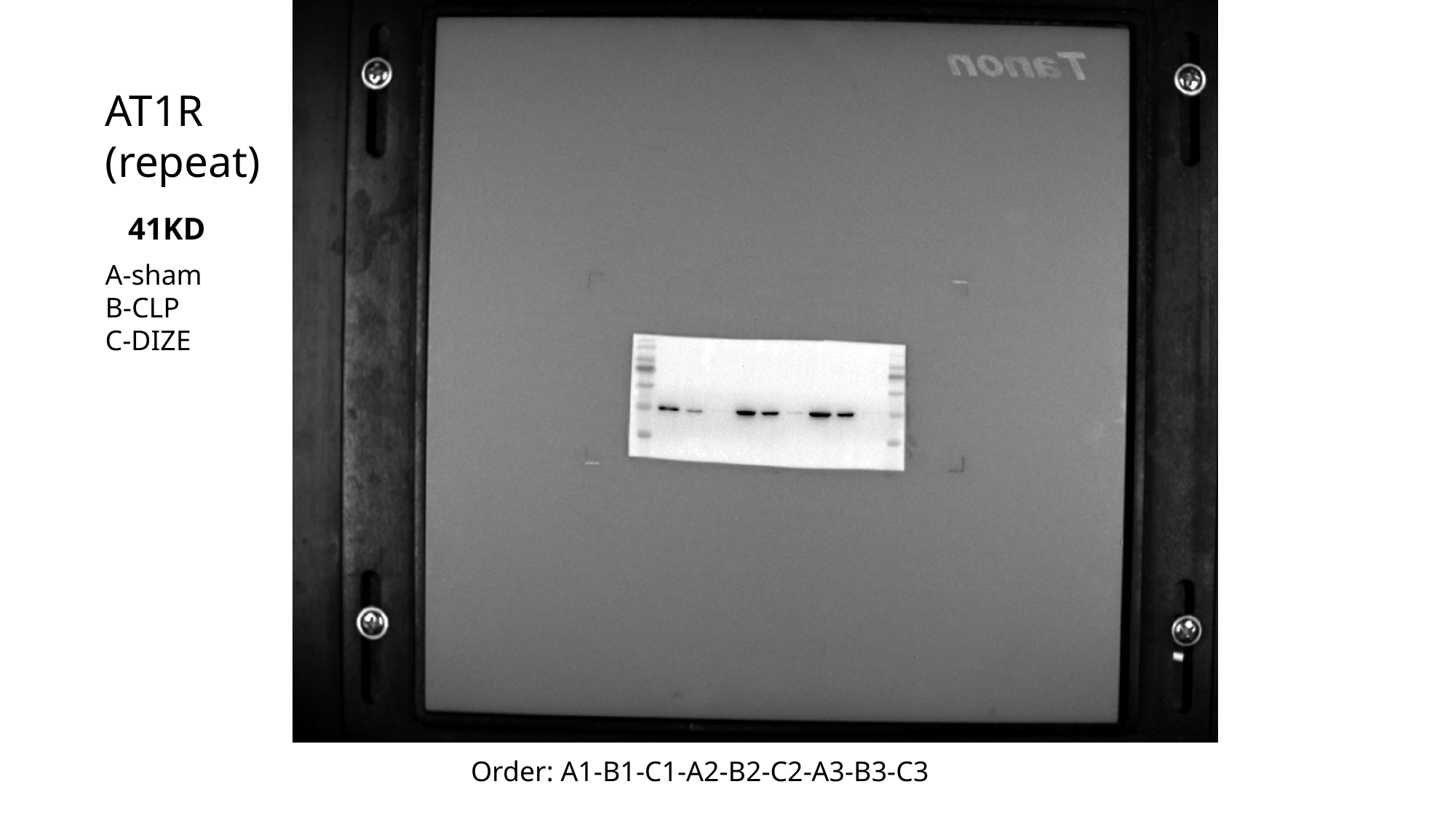

AT1R (repeat)
41KD
A-sham
B-CLP
C-DIZE
Order: A1-B1-C1-A2-B2-C2-A3-B3-C3

## Slide 12
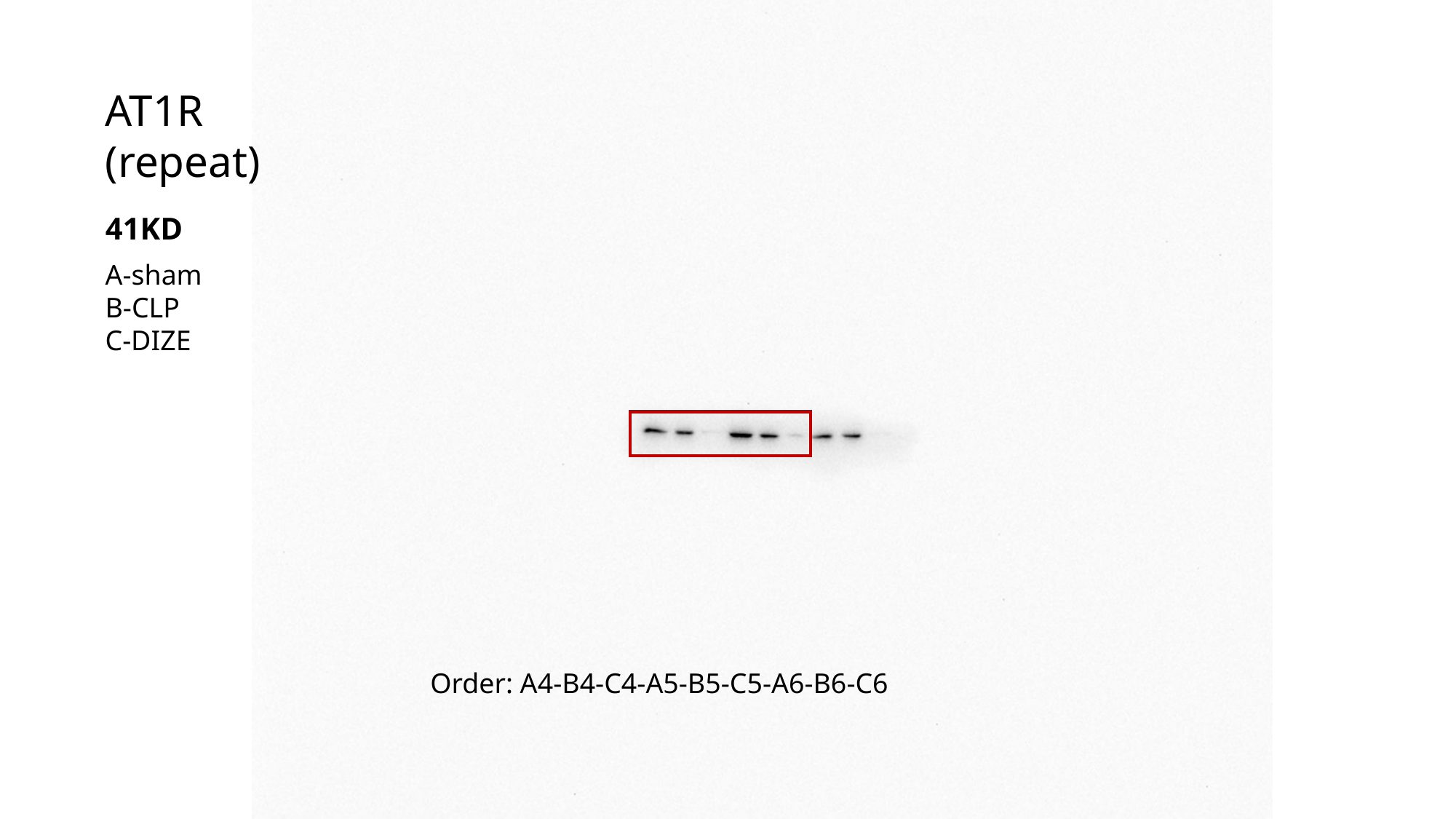

AT1R (repeat)
41KD
A-sham
B-CLP
C-DIZE
Order: A4-B4-C4-A5-B5-C5-A6-B6-C6

## Slide 13
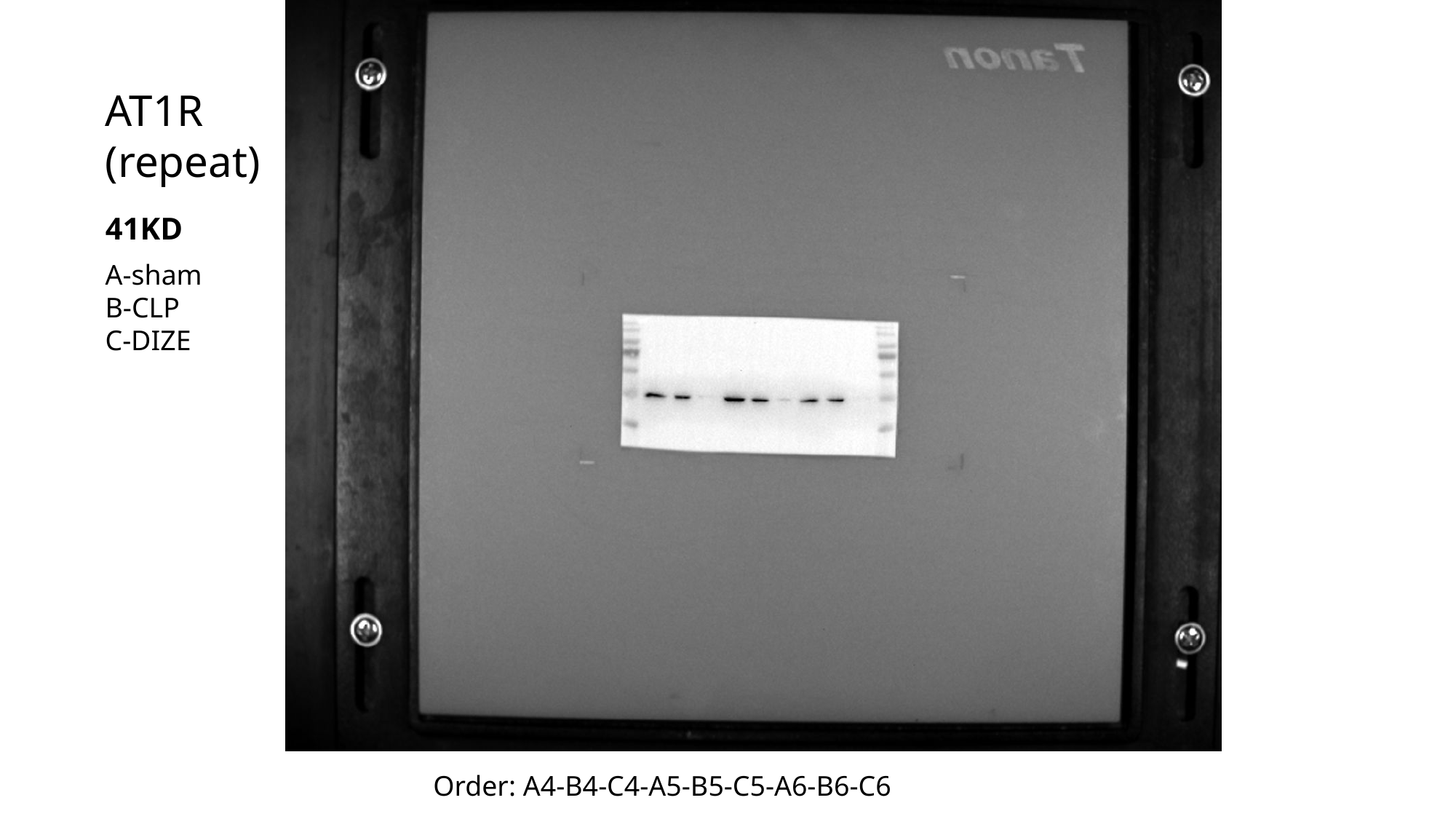

AT1R (repeat)
41KD
A-sham
B-CLP
C-DIZE
Order: A4-B4-C4-A5-B5-C5-A6-B6-C6

## Slide 14
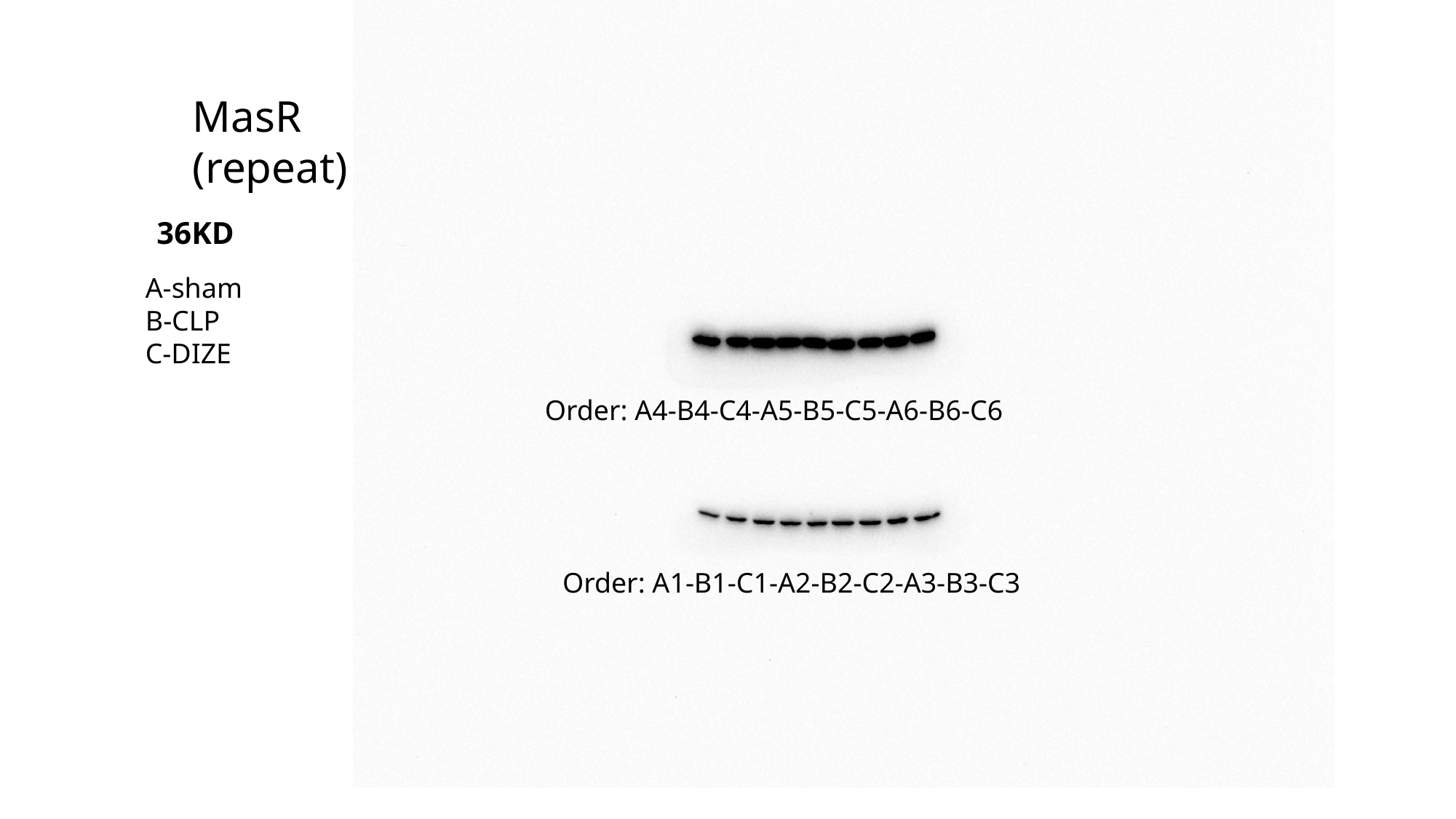

MasR (repeat)
36KD
A-sham
B-CLP
C-DIZE
Order: A4-B4-C4-A5-B5-C5-A6-B6-C6
Order: A1-B1-C1-A2-B2-C2-A3-B3-C3

## Slide 15
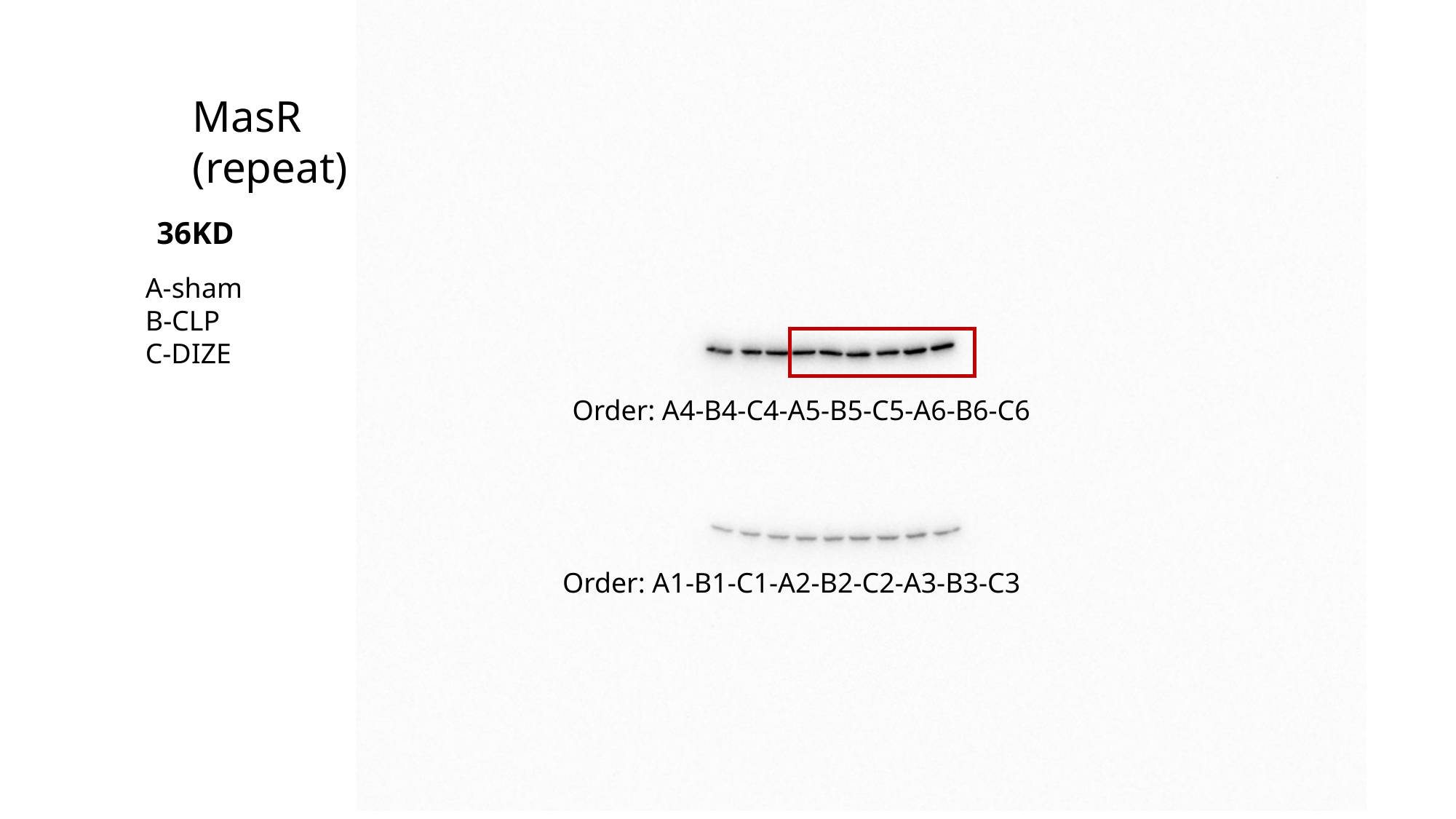

MasR (repeat)
36KD
A-sham
B-CLP
C-DIZE
Order: A4-B4-C4-A5-B5-C5-A6-B6-C6
Order: A1-B1-C1-A2-B2-C2-A3-B3-C3

## Slide 16
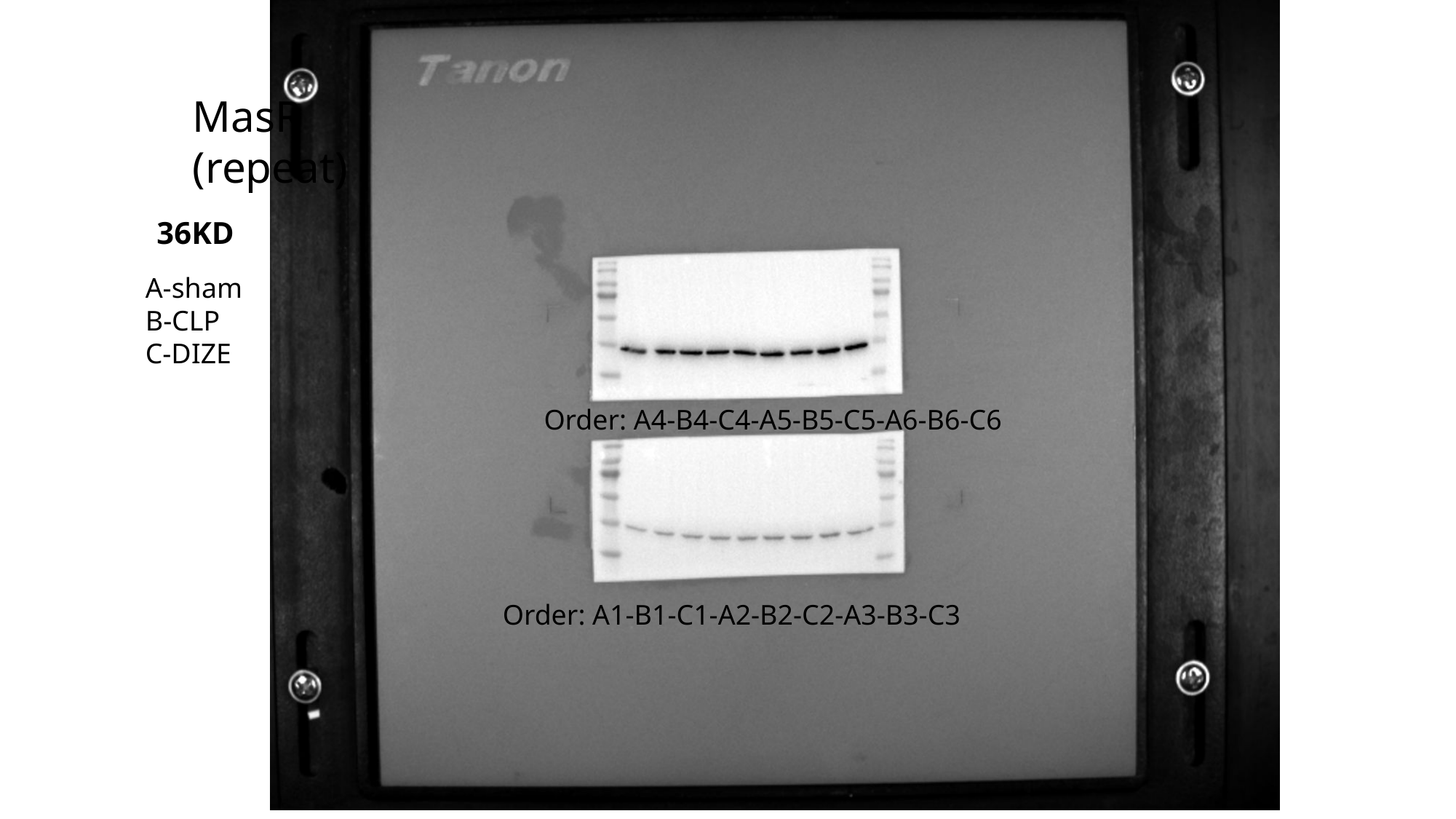

MasR (repeat)
36KD
A-sham
B-CLP
C-DIZE
Order: A4-B4-C4-A5-B5-C5-A6-B6-C6
Order: A1-B1-C1-A2-B2-C2-A3-B3-C3

## Slide 17
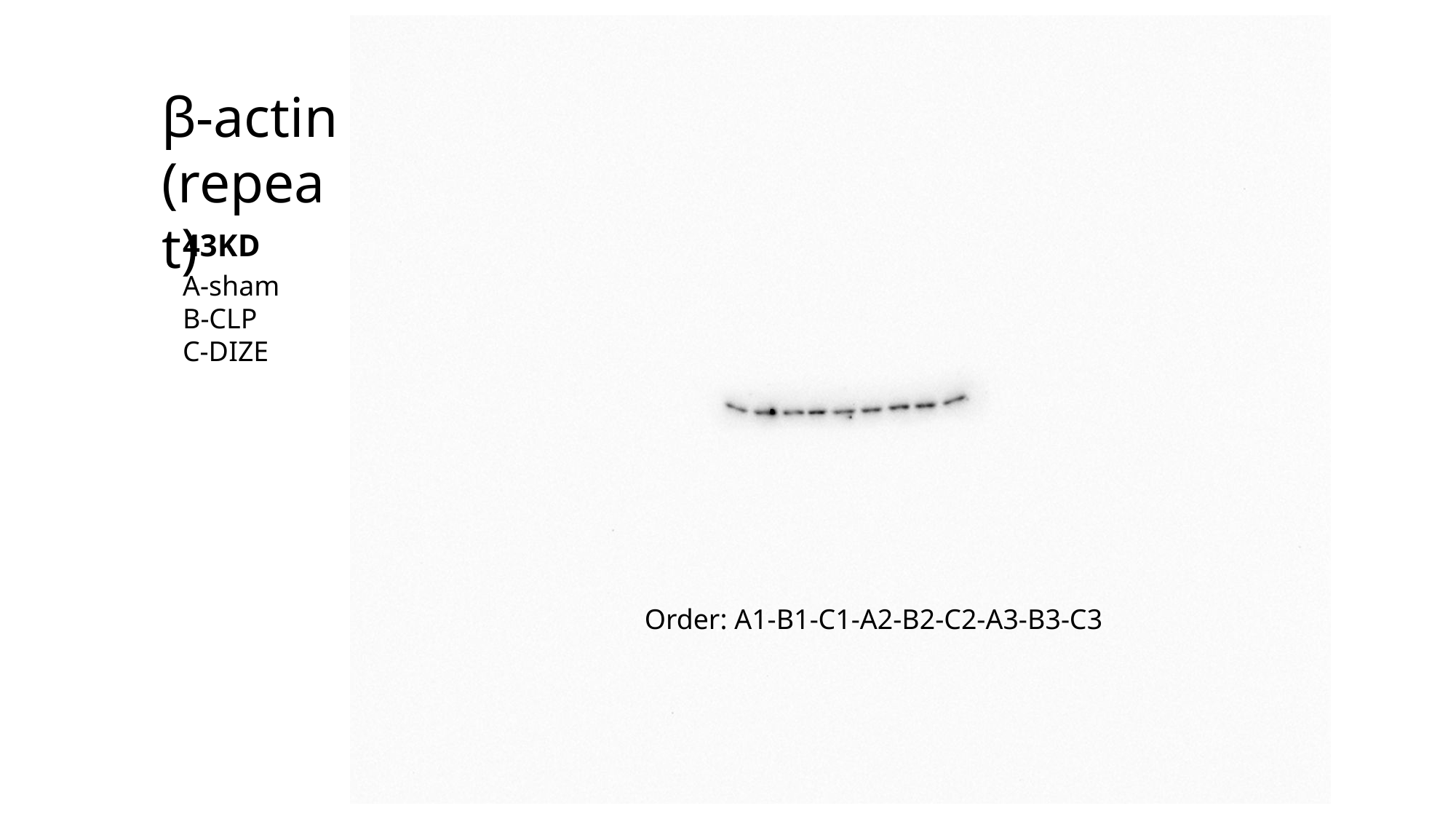

β-actin (repeat)
43KD
A-sham
B-CLP
C-DIZE
Order: A1-B1-C1-A2-B2-C2-A3-B3-C3

## Slide 18
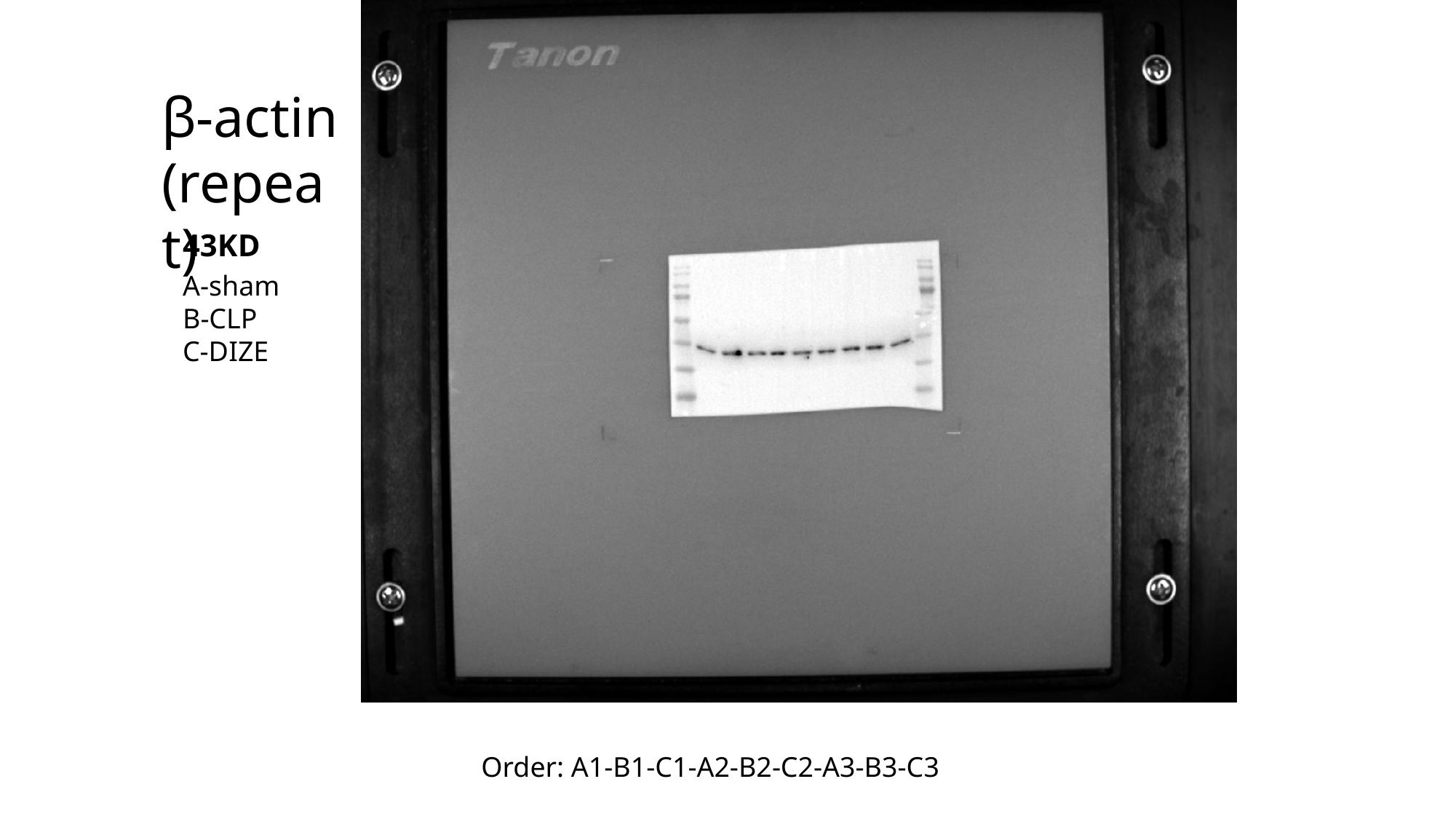

β-actin (repeat)
43KD
A-sham
B-CLP
C-DIZE
Order: A1-B1-C1-A2-B2-C2-A3-B3-C3

## Slide 19
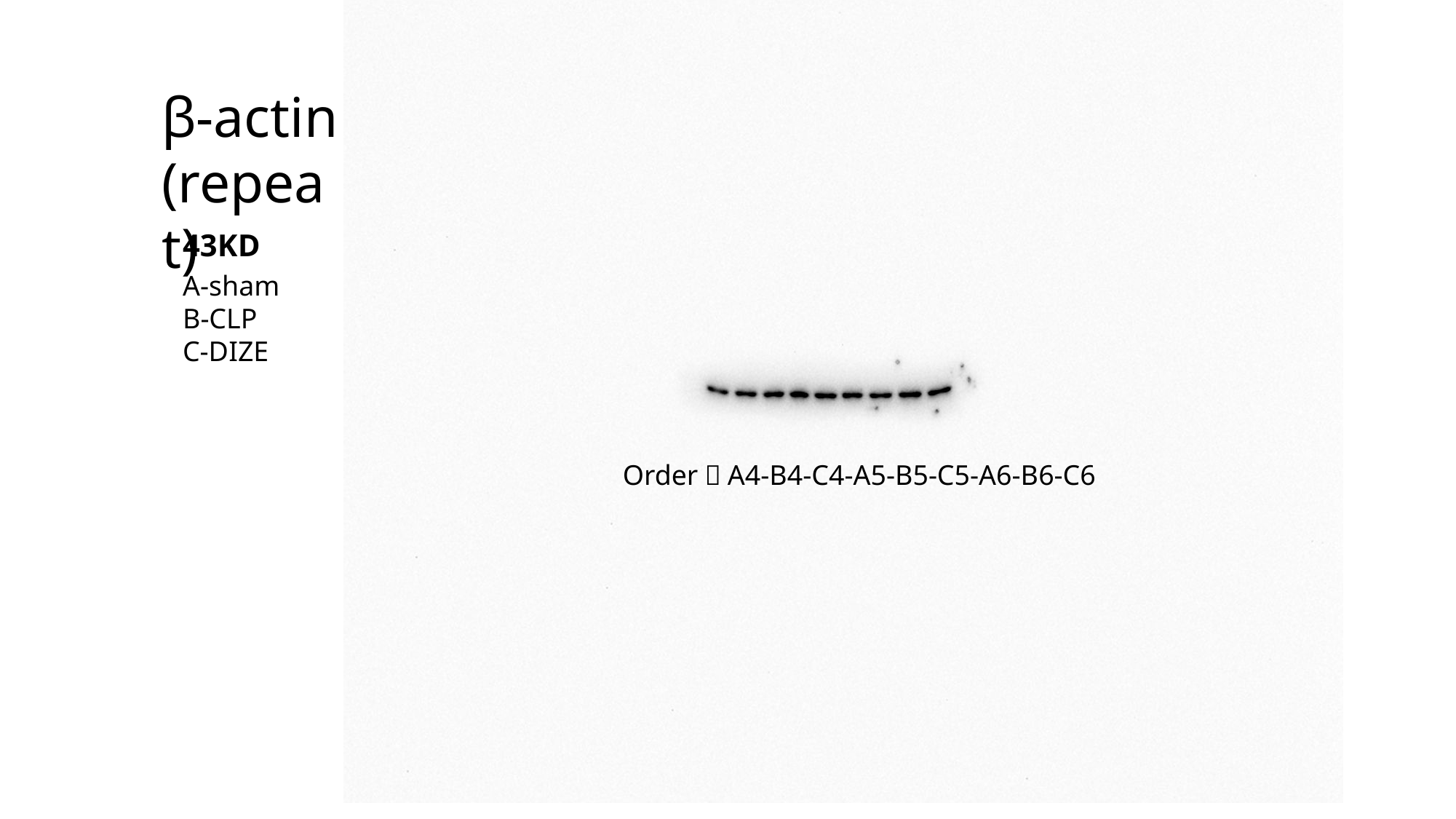

β-actin (repeat)
43KD
A-sham
B-CLP
C-DIZE
Order：A4-B4-C4-A5-B5-C5-A6-B6-C6

## Slide 20
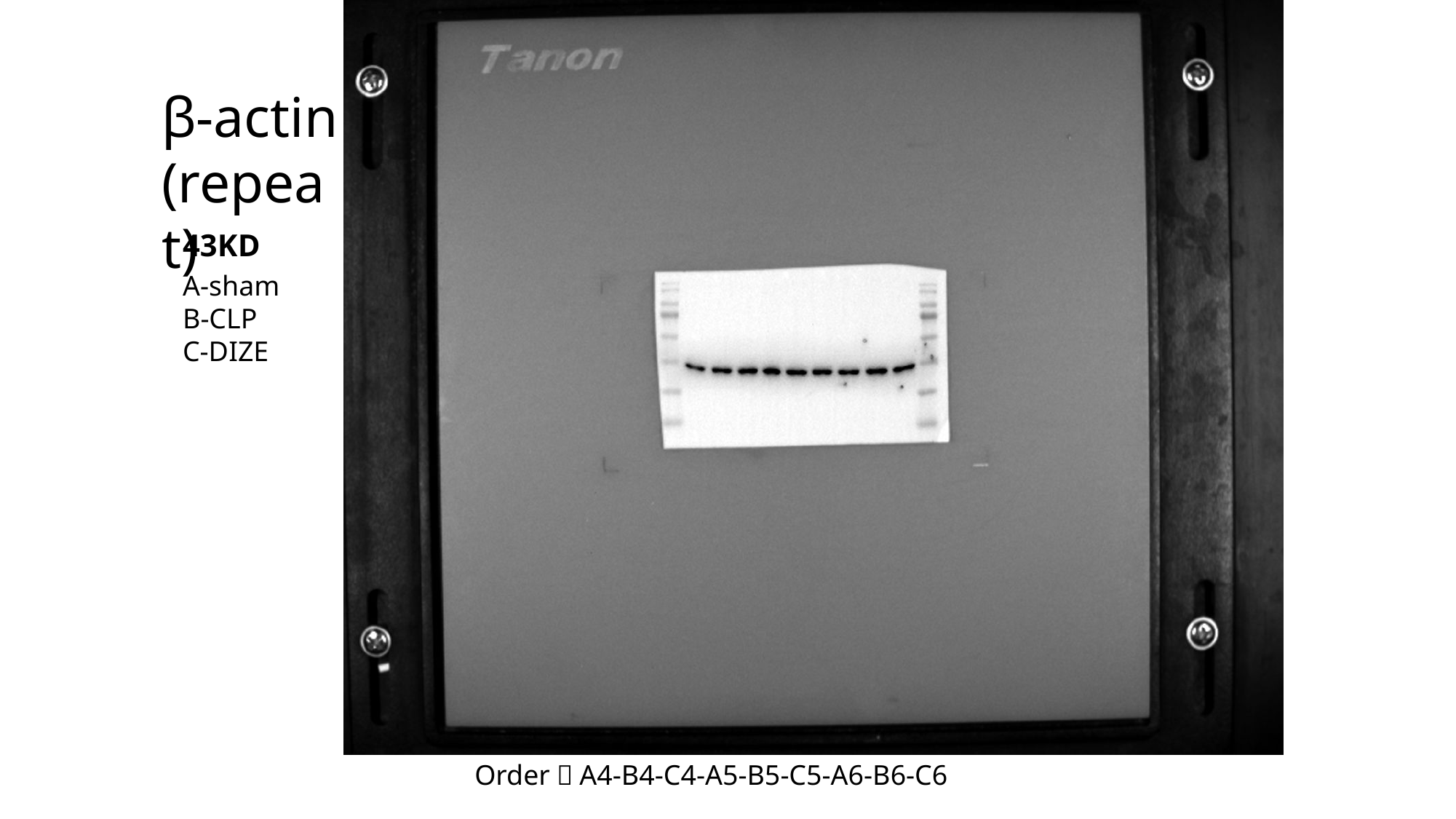

β-actin (repeat)
43KD
A-sham
B-CLP
C-DIZE
Order：A4-B4-C4-A5-B5-C5-A6-B6-C6
